# Development and validation of dynamic clinical subphenotypes in acute pancreatitis patients using vital sign trajectories in intensive care units: a multinational cohort study

**DOI:** 10.1038/s41392-025-02261-4

**Published:** 2025-06-04

**Authors:** Zichen Wang, Wen Wang, Jiayue Xu, Qiao He, Che Sun, Shuangyi Xie, Kang Zou, Qing Xia, Xin Sun

**Affiliations:** 1 Institute of Integrated Traditional Chinese and Western Medicine, Chinese Evidence-based Medicine Center and Cochrane China, West China Hospital, Sichuan University, Chengdu, Sichuan China; 2 NMPA Key Laboratory for Real World Data Research and Evaluation in Hainan, Chengdu, 610041 China; 3 Sichuan Center of Technology Innovation for Real World Data, Chengdu, China; 4 West China School of Public Health and West China Fourth Hospital, Sichuan University, Chengdu, China

**Keywords:** Medical research, Gastrointestinal diseases

## Abstract

Acute pancreatitis (AP) is a heterogeneous inflammatory condition. Although emerging therapeutic strategies targeting pathways such as calcium signaling, TNF-α, the NLRP3 inflammasome, and HMGB1 have shown promise, their efficacy may be limited by the underlying biological heterogeneity of the disease. In this multinational retrospective cohort study across three large ICU databases (ICU-HAI, MIMIC-IV, and eICU-CRD), we used group-based trajectory modeling of early vital signs to identify four distinct AP subphenotypes: hyperinflammatory, hypertensive, hypotensive, and hypoinflammatory. These subtypes differed markedly in 30-day mortality, inflammatory burden, and hemodynamic stability. Compared to the hypertensive phenotype, hyperinflammatory and hypotensive patients had significantly higher 30-day mortality risks (HR = 3.38 and HR = 1.87, respectively), while the hypoinflammatory phenotype carried no excess risk. Fluid resuscitation responses were phenotype-specific: hyperinflammatory patients benefited from higher fluid volumes, whereas hypoinflammatory patients were at risk of fluid overload. Notably, distinct subphenotypes displayed unique responses to fluid intake over the first two ICU days. For hyperinflammatory phenotype, the algorithm-estimated lowest-risk fluid range was 4100–4300 mL on day 1 and 3400–3600 mL on day 2; for phenotype hypoinflammatory phenotype, the optimal ranges were 2800–3800 mL and 1400–2600 mL, respectively. Early use of lactated Ringer’s solution, which inhibited NLRP3, was associated with reduced mortality in hypotensive phenotype. These findings underscore the clinical relevance of early physiological trajectories and support precision fluid resuscitation based on subtype. This study establishes the largest early-trajectory-based classification of AP to date, offering new insights into immune and vascular mechanisms that drive heterogeneity and therapeutic responsiveness.

## Introduction

Acute pancreatitis (AP) is a multifaceted emergency inflammatory condition and is the leading gastrointestinal disease that causes hospitalization, with a continually increasing global annual incidence rate.^1–4^ Recent global estimates report an incidence of 33.7 cases and 1.6 deaths per 100,000 individuals annually, highlighting its significant epidemiological footprint.^1,5^ A comprehensive meta-analysis by Iannuzzi et al. examined trends from 1961 to 2016 across 44 studies, revealing a significant global increase in AP incidence, with a pooled average annual percent change (AAPC) of 3.07%.^3^ This trend mirrors the rise in disability-adjusted life-years attributable to AP, which climbed from 2.4 million in 1990 to 3.6 million in 2019, reflecting a mounting burden on global health systems.^4^ In the United States, the incidence of AP ranges between 110 and 140 cases per 100,000 population annually, contributing to over 300,000 hospital admissions each year, with an average direct healthcare cost exceeding USD 10,000 per patient.^1,6^ Approximately 20% of AP patients progress to severe AP(SAP), characterized by persistent organ failure that requires intensive care unit (ICU) admission, resulting in a mortality rate of 20–40%.^7–11^ Despite the high clinical burden, advances in understanding AP pathogenesis and therapeutic intervention remain limited.^1,2,12^

Recent mechanistic studies have demonstrated that the pathogenesis of AP involves a complex network of interconnected cellular processes. Core pathological events include the premature activation of trypsinogen within pancreatic acinar cells,^13–16^ endoplasmic reticulum stress,^17,18^ dysregulation of the unfolded protein response,^19,20^ intracellular Ca²⁺ overload, and impaired autophagy.^21,22^ These processes synergistically contribute to acinar cell injury and death, triggering local inflammation and promoting systemic immune responses. Accumulating evidence suggests that AP has significant inter-individual variability in disease mechanisms, clinical phenotypes, and treatment responses. Such heterogeneity may be shaped by genetic diversity,^23^ etiological factors, and patients' individualized conditions at disease onset, which together result in diverse molecular profiles across transcriptomic, proteomic, and metabolomic levels.^24^ These observations highlight the limitations of uniform treatment strategies and underscore the need for therapeutic approaches that are tailored to the individual’s specific pathophysiological traits. A growing body of preclinical and early-phase clinical studies has identified a diverse array of therapeutic candidates targeting distinct molecular pathways, further underscoring the complexity of its pathophysiology. These findings suggest that effective interventions may require pathway-specific targeted therapies rather than uniform treatment strategies, highlighting the need to align therapeutic approaches with the individual’s underlying disease mechanisms. For example, CM4620 (Auxora), a selective store-operated calcium entry inhibitor, and GsMTx4, a Piezo1 inhibitor, effectively attenuate Ca²⁺ overload and tissue injury in experimental models. Similarly, caffeine Gut (an inositol 1,4,5-trisphosphate receptor antagonist) and dantrolene (a ryanodine receptor inhibitor) have shown protective effects through modulation of calcium signaling and reduction of acinar cell damage.^25^ Beyond calcium-targeting agents, immunomodulators such as infliximab (anti–TNF-α monoclonal antibody),^26^ CXCL10-neutralizing antibodies acting downstream of MLKL signaling,^27^ and lactate-mediated inhibition of the NLRP3 inflammasome^25^ have demonstrated potential in suppressing systemic inflammatory responses. Additional agents—including heparin derivatives neutralizing extracellular HMGB1 and dabigatran, a dual inhibitor of proteases and coagulation pathways may mitigate disease progression and systemic injury.^25,28^ Adjunctive approaches, such as natural compounds targeting NF-κB and MAPK signaling^29^ and acupuncture interventions that enhance gastrointestinal motility and symptom relief,^30^ are also under investigation.

Despite these advances, treatment outcomes may still likely be affected by the inherent biological heterogeneity of AP. While clinically defined as a single disease, AP encompasses diverse molecular drivers—including variations in inflammatory signaling and immune activation—that can differ significantly between individuals. This mechanistic diversity corresponds with a broad clinical spectrum, ranging from localized pancreatic inflammation to systemic multiorgan failure. Clinical features such as hyperthermia, hypothermia, tachycardia, hypoperfusion, azotemia, and metabolic derangements are frequently observed and are essential for early risk assessment and prognostic evaluation. Given the variability in both pathophysiology and clinical presentation, there is a pressing need to delineate clinically meaningful subgroups within the AP population to support individualized therapeutic decisions.^7,31–34^ However, the current studies on AP subtyping were limited. A pivotal study by Neyton et al., leveraging dynamic multi-omics, has elucidated the diverse molecular patterns within AP, hinting at its potential clinical subphenotypes.^24^ Yet, the study’s reliance on less commonly used omics profiling and not establishing links between subtypes and specific treatments limits its generalizability and clinical applicability.

In ICU settings, routine monitoring of vital signs offers dynamic insights into patient status, thereby facilitating stratified patient inclusion in clinical studies.^35,36^ Vital sign trajectories embodying multidimensionality and dynamism have been instrumental in sepsis subphenotyping, providing critical insights into disease evolution and individualized treatment plans.^36^ Inspired by the above evidence and driven by the growing recognition of biological heterogeneity as well as the urgent need for precision-guided therapies in AP, we initiated a multinational retrospective cohort study across three large ICU databases from China and the United States. This study was specifically designed to identify early subphenotypes of AP that are both clinically and biologically meaningful, by leveraging dynamic trajectories of vital signs recorded within the first 12 h following ICU admission. This early time frame reflects a critical period during which systemic physiological responses emerge and initial therapeutic interventions are most likely to influence disease progression. Our study was structured around three key objectives. First, we aimed to develop and validate robust, trajectory-defined AP subphenotypes using unsupervised machine learning techniques grounded in early physiological data. Second, we sought to comprehensively characterize these subphenotypes in terms of clinical presentation, illness severity, and prognostic stratification, providing surrogate indicators of potential underlying pathobiological mechanisms. Third, we planned to explore the heterogeneity of treatment effects—focusing particularly on the differential associations between early fluid resuscitation strategies and outcomes across subphenotypes.

## Result

### Baseline characteristics

**Fig. 1:**
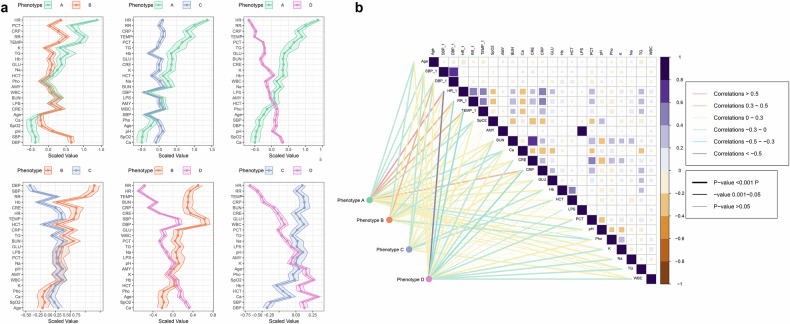
Clinical characteristics among different subphenotypes. **a** Illustrates the variation in baseline vital signs and laboratory test values across AP subphenotypes. These values have been standardized using Z-scores for clarity. Each sub-plot presents a comparison between two phenotypes; **b** shows the Pearson correlation between variables and subphenotypes. In the right plot, the triangular section displays the correlations between variables. Darker (purple) squares indicate positive correlations, while lighter (yellow) squares indicate negative correlations. The size of each square corresponds to the absolute value of the correlation coefficient. The lower-left section depicts the correlation between phenotypes and clinical characteristics. The color represents the correlation coefficients, while the width indicates the significance of the correlation. Patient data from the development cohort were used for data analysis in this figure

A total of 3080 AP patients were included from 2011 to 2021 in databases of China and the United States (Supplementary Fig. 1). Characteristics and clinical outcomes of developed subphenotypes were compared (Table 1, Fig. 1a, b, and Supplementary Tables 1–3). Phenotype A exhibited elevated levels of amylase, lipase, BUN, creatinine, sodium, potassium, CRP, PCT, glucose, triglycerides, and the highest proportion of congestive heart failure and renal disease, categorizing it as the “hyperinflammatory subphenotype”. Phenotype B displayed the highest baseline blood pressure (denoted as the “hypertensive subphenotype”), while phenotype C had the lowest baseline blood pressure, amylase, Hb, and hematocrit (denoted as the “hypotensive subphenotype”). Phenotype D showed the most aged and had the lowest heart rate, RR, TEMP, lipase, BUN, creatinine, WBC, CRP, PCT, glucose, triglycerides, and the highest baseline oxygen saturation and Hb and was characterized as “hypoinflammatory subphenotype” (Table 1, Fig. 1a, and Supplementary Table 1).

**Table 1:** The clinical characteristics of subphenotypes in development cohort

Phenotype	Phenotype A	Phenotype B	Phenotype C	Phenotype D	*p*
N	369	461	706	1076	
Age	45.0 (35.0, 53.0)	45.0 (36.0, 52.0)	48.0 (39.0, 57.0)	49.0 (41.0, 60.0)	<0.001
Gender					
Male	235 (63.7)	329 (71.4)	457 (64.7)	720 (66.9)	0.064
Female	134 (36.3)	132 (28.6)	249 (35.3)	356 (33.1)	
Race					
Asia	369 (100.0)	461 (100.0)	706 (100.0)	1076 (100.0)	/
Myocardial infarction (%)	0 (0.0)	1 (0.2)	2 (0.3)	3 (0.3)	0.788
Congestive heart failure (%)	33 (8.9)	28 (6.1)	57 (8.1)	53 (4.9)	0.012
Cerebrovascular disease (%)	14 (3.8)	16 (3.5)	24 (3.4)	31 (2.9)	0.817
Chronic pulmonary disease (%)	11 (3.0)	17 (3.7)	22 (3.1)	72 (6.7)	0.001
Diabetes (%)	85 (23.0)	100 (21.7)	149 (21.1)	218 (20.3)	0.710
Liver disease (%)	169 (46)	207 (44.9)	284 (40.2)	485 (45.1)	0.158
Renal disease (%)	41 (11.1)	36 (7.8)	66 (9.3)	54 (5.0)	<0.001
Malignant tumor (%)	12 (3.3)	14 (3.0)	24 (3.4)	146 (13.6)	<0.001
ICU mortality (%)	46 (12.5)	23 (5.0)	47 (6.7)	22 (2.0)	<0.001
Hospital mortality (%)	46 (12.5)	24 (5.2)	51 (7.2)	27 (2.5)	<0.001
Hospital length of stay (day)	25.0 (14.0, 39.0)	25.0 (16.0, 37.0)	23.0 (14.0, 37.0)	18.0 (12.0, 27.0)	<0.001
ICU length of stay (day)	10.0 (5.00, 20.0)	9.0 (4.0, 17.0)	7.0 (4.0, 14.0)	3.0 (2.0, 5.0)	<0.001

### Vital sign trajectories and derivation of AP subphenotypes

The visual trajectory of vital signs 12 h after ICU admission of AP patients in the development cohort was shown (Supplementary Fig. 2). For each patient, vital signs exhibited intricate fluctuations, with numerous intersections observed in the trajectory of each patient’s vital signs that indicated a notable variability in the disease status among patients with AP.

The application of consensus clustering successfully determined that K = 4 represented the optimal choice for characterizing subphenotypes within AP (Supplementary Figs. 3–5). Sensitivity analyses involving model matrices of GBMTMs for K = 2 to 6 consistently corroborated these findings (Supplementary Table 4). Therefore, GBMTM with 4 subclasses was identified as the final model (Supplementary Table 5). Further analysis demonstrated that using 9-h trajectories can achieve 91.21% accuracy for early phenotype predictions (Supplementary Fig. 6).

**Fig. 2:**
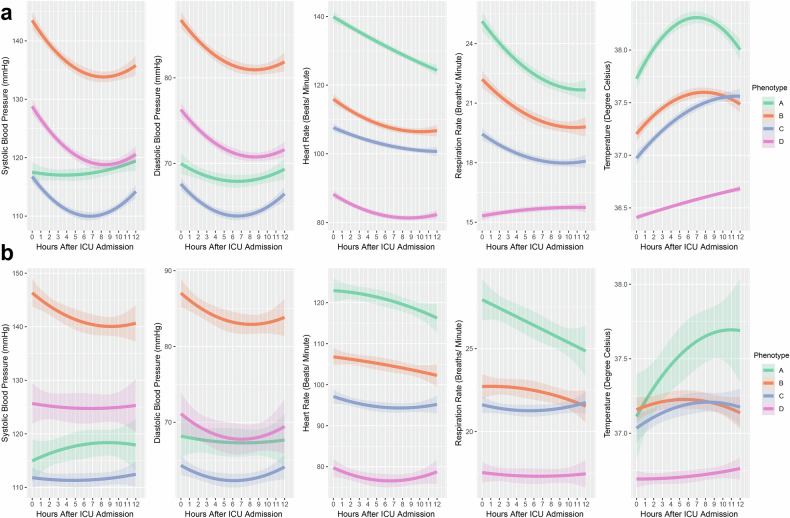
Group-based trajectory modeling of vital signs in the development and validation cohorts. AMY amylase, CRE creatinine, GLU glucose, HCT hematocrit, LPS lipase, Pho phosphate, K potassium, Na sodium, TG triglycerides, WBC white blood cell; **a** displays the group-based vital signs in the development cohort, while (**b**) shows the group-based vital signs in the validation cohort. The shading represents the 95% confidence intervals for each vital sign trajectory

Within the development and validation cohorts, phenotype A (14.1 and 16.0%, respectively) was characterized by hyperthermia, tachycardia, and tachypnea. Phenotype B (17.6 and 30.0%, respectively) exhibited hypertension. Phenotype C (27.0 and 31.9%, respectively) displayed hypotensive, while phenotype D (41.2 and 24.1%, respectively) exhibited hypothermia, bradycardia and bradypnea (Fig. 2). Differences in vital signs distribution between phenotypes were also evident in the temporal data (Supplementary Fig. 7).

### Clinical outcomes across subphenotypes

Phenotypes A exhibited the highest ICU/in-hospital mortality rate, and the longest median ICU/ hospital LOS, while phenotypes D exhibited the lowest ICU/in-hospital mortality rate and the shortest median ICU/hospital LOS. We also illustrated the correlation across baseline variables and the correlation between variables and subphenotypes (Fig. 1b).

**Fig. 3:**
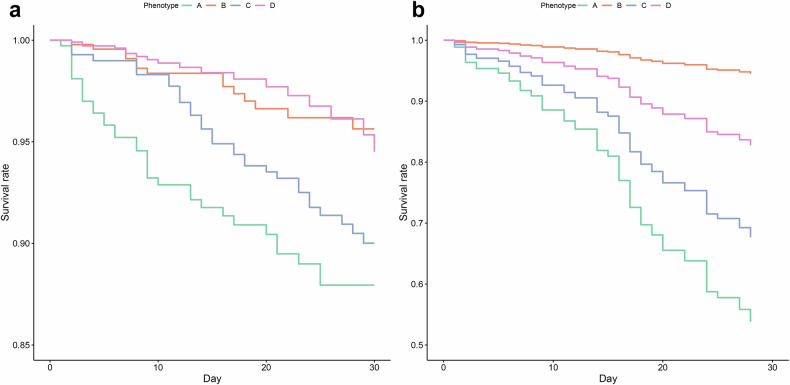
The Kaplan–Meier of 30-day mortality for subphenotypes in development and validation cohorts. **a** represented the KM curve of 30-day mortality across AP subphenotypes in the development cohort, while (**b**) represented the KM curve of 30-day mortality across AP subphenotypes in the validation cohort; KM curves were adjusted by demographic characteristics (age, gender, and race)

Adjusted Kaplan–Meier plots illustrated a consistent trend in 30-day prognosis for AP subphenotypes in both the development and validation cohorts. Compared to phenotype B, phenotypes A and C exhibited a higher risk of 30-day mortality, while there was no significant difference in prognosis between phenotype B and phenotype D (Fig. 3a, b).

In the development cohort, Cox regression analysis further supported these findings. In comparison to phenotype B, the crude HR of phenotype A [HR 3.17, 95% confidence interval (CI): 1.71–5.89, *p* < 0.001] and Phenotype C (HR 2.12, 95% CI: 1.16-3.90, *p* = 0.015) showed a higher mortality risk, while no significant difference in 30-day mortality risk was observed in phenotype D (HR 0.94, 95% CI: 0.47–1.90 *p* = 0.865). After adjustment for demographics, Phenotype A [HR 3.38, 95% CI: 1.82–6.29, *p* < 0.001] and phenotype C (HR 1.87, 95% CI: 1.02–3.45, *p* = 0.043) also showed higher mortality risk than phenotype B, and no significant difference in 30-day mortality risk was observed for phenotype D (HR 0.77, 95% CI: 0.38–1.56, *p* = 0.466). External validation results showed a consistent trend, with phenotypes A and C demonstrating a higher risk before and after adjusting for demographic characteristics, while phenotype D showed no significant difference in 30-day mortality (Table 2). Multivariate Cox regression revealed that chronic pulmonary disease significantly increased mortality risk in phenotypes B and C, while renal disease elevated risk in phenotypes C and D (Supplementary Table 6).

**Table 2:** Cox regression result between subphenotypes of 30-day mortality in development and validation cohorts Multivariate Cox regression were adjusted by demographic characteristics (age, gender, race)

Development cohort (ICU-HAIs registry)							
	Phenotype B	Phenotype A		Phenotype C		Phenotype D	
		HR (95%CI)	*P* value	HR (95%CI)	*P* value	HR (95%CI)	*P* value
30-day mortality							
Crude	Reference	3.17 (1.71,5.89)	<0.001	2.12 (1.16.3.90)	0.015	0.94 (0.47,1.90)	0.865
Adjusted	Reference	3.38 (1.82,6.29)	<0.001	1.87 (1.02,3.45)	0.043	0.77 (0.38,1.56)	0.466
External validation cohort (MIMIC-IV, eICU-CRD)							
30-day mortality							
Crude	Reference	13.39 (1.72,103.99)	0.013	11.68 (1.56,87.23)	0.017	7.12 (0.83,60.99)	0.073
Adjusted	Reference	15.21 (1.95,118.68)	0.009	9.57 (1.27,72.20)	0.002	1.65 (0.59,45.990)	0.138

### Heterogeneity of treatment responses of fluid intake within 48 h of ICU admission

**Fig. 4:**
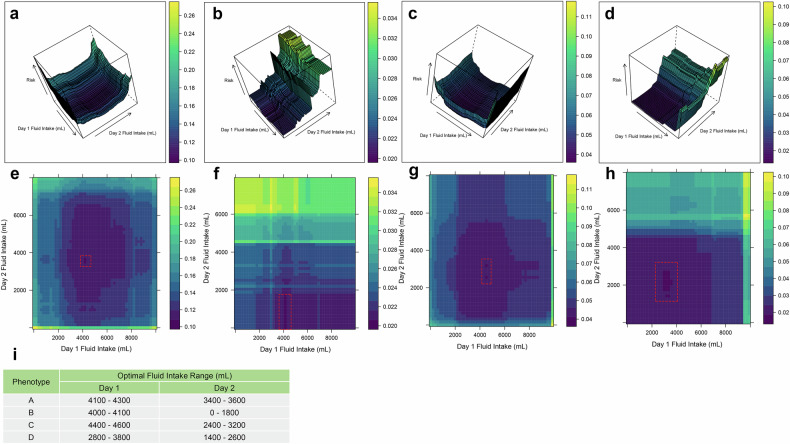
Partial dependence plots for distinct random forest models fitted for subphenotypes. **a**–**d** represented the 3D visualization between 1st and 2nd day fluid intake and ICU mortality among AP subphenotypes; **e**–**h** represented the 2D heat map visualization between 1st and 2nd day fluid intake and ICU mortality among AP subphenotypes

An RF-based ICU death classifier was developed for the four AP subphenotypes, which accurately discriminated between all mortality and survival cases A RF-based ICU death classifier was developed for the four AP subphenotypes (Supplementary Fig. 8). Additionally, employing the PDP algorithm, we visualized the relationship between the first 48 hours of fluid intake volumes (summarized daily) and the risk of ICU mortality (Fig. 4a–h). These 3D representations highlight distinct response patterns among the subphenotypes based on varying fluid replenishment volumes (Fig. 4a–d). Notably, for phenotype B, ICU fluid resuscitation on the first day has minimal impact on mortality, but when fluid intake is over 4000 mL on the following day significantly elevates the risk of ICU death. Conversely, for phenotype D, the degree of risk is influenced by both the first and the next day’s replenishment. Stratifying according to risk gradients, we identified PDP-calculated fluid rehydration ranges associated with the lowest mortality risk for different AP subphenotypes (depicted in dark purple) (A: Day1 4100–4300, Day2 3400–3600; B: Day1 4000–4100, Day2 0–1800; C: Day1 4400–4600, Day2 2400–3200; D: Day1 2800–3800, Day2 1400–2600) (Fig. 4i). Multivariate Cox regression revealed that in phenotype C, patients who received lactated Ringer’s solution within 48 h showed reduced 30-day mortality risk compared to patients who did not (HR: 0.48, 95% CI: 0.28–0.82) (Supplementary Table 9).

## Discussion

In this study, we developed and validated four robust subphenotypes (phenotype A: hyperinflammatory, phenotype B: hypertensive, phenotype C: hypotensive, and phenotype D: hypoinflammatory) of AP using 12 h vital sign trajectories after ICU admission. We also explored the heterogeneity of treatment response to fluid intake within 48 h of ICU admission. In phenotype B, ICU fluid resuscitation volume on the first day had an insensitive impact on mortality, while fluid intake >4000 mL on the following day significantly increased the risk of ICU death. In contrast, in phenotype D, the risk was influenced by both the first day and the next day of resuscitation. We also explored PDP-calculated lowest-risk ranges of fluid resuscitation in different AP subphenotypes. Our findings indicated that AP subphenotypes exhibit significant differences in clinical characteristics and prognosis. Phenotype A exhibited hyperthermia, tachycardia, and tachypnea, accompanied by elevated CRP and PCT levels, and displayed the highest 30-day mortality risk, which represents a hyperinflammatory condition. In contrast, phenotype D, with higher age and lower TEMP, heart rate, RR, CRP, and PCT levels, showed a relatively lower mortality risk, which represents a hypoinflammatory condition.^22,37^ Inflammation is central to AP pathophysiology, with dynamic and patient-specific responses shaping disease severity and outcomes. Aberrant pancreatic enzyme activation triggers NF-κB-mediated inflammatory cascades, leading to the sequential release of pro- and anti-inflammatory mediators (e.g., IL-6, IL-8, MCP-1; HGF, and sTNF-αR1) with severe cases show an early pro-inflammatory surge, while anti-inflammatory mediators dominate later stages.^38,39^ This heterogeneity in inflammatory phenotypes affects immune cell infiltration in the pancreas and is closely associated with organ failure and complications, thus accurate identification of inflammatory status is crucial for personalized treatment of AP.

Interestingly, similar to our findings, Neyton et al.^24^ also identified four AP endotypes with distinct molecular patterns. Endotype A, representing a hyperinflammatory state, was associated with the highest disease severity, systemic inflammatory response syndrome (SIRS) prevalence, and mortality, indicating excessive immune activation. In contrast, Endotype C, representing a hypoinflammatory state, had a SIRS prevalence of 0% and exhibited the lowest disease severity, further validating the heterogeneity of inflammatory responses in AP. Their study^24^ also identified molecular markers associated with these inflammatory endotypes: hyperinflammatory Endotype A was linked to upregulation of *N*-acetyl-3-methylhistidine, *N*-acetyl-1-methylhistidine, XIRP1, and MAP3K6, whereas hypoinflammatory Endotype C was associated with increased levels of GGT2 (γ-glutamyltransferase 2), dopamine sulfate, citrulline, and SPTSSB. These molecules may serve as specific targets reflecting inflammatory heterogeneity.

Using machine learning and PDP algorithms, our study further demonstrated that phenotypes A–D exhibit distinct responses to fluid resuscitation strategies. Phenotype B was sensitive to fluid intake volume, especially on the second day, which may be related to its initial hypertensive state. In phenotype C, early administration of lactated Ringer’s solution during initial resuscitation was significantly associated with improved 30-day survival. Lactate, a key component of this balanced crystalloid, has been shown to attenuate inflammation by inhibiting NLRP3 inflammasome activation and IL-1β production via the TLR4 pathway,^40,41^, which suggests that targeting NLRP3-mediated inflammatory pathways may confer benefit in patients with specific disease mechanisms. The observed association further supports the hypothesis that therapeutic efficacy may depend on the underlying biological state, highlighting the importance of tailoring interventions to distinct clinical subphenotypes. Notably, phenotypes A and D exhibited the highest and lowest PDP-calculated lowest-risk ranges, respectively, potentially reflecting differences in hyperinflammatory versus hypoinflammatory pathway activation. Our observation aligns with findings by Zhang et al. in septic shock patients, where low-inflammation endotypes benefited more from restrictive fluid resuscitation, whereas high-inflammation endotypes demonstrated the opposite trend.^42^ The above findings suggested that clinical characteristics and underlying molecular patterns influence patient responses to fluid resuscitation strategies. The distinct molecular signatures and individualized treatment responses of the AP subphenotypes developed in our study provide valuable insights for future research on targeted therapies and personalized treatment strategies based on specific pathways and therapeutic targets. In future work, we will focus on prospectively validating the stability of the four AP subphenotypes identified in retrospective analyses, assessing whether their trajectory patterns, clinical characteristics, prognostic trends, and responses to fluid therapy remain consistent over time. Beyond this validation, a key objective will be to elucidate the underlying molecular mechanisms of these subphenotypes through multi-omics analyses, aiming to establish their correspondence with distinct endotypes. By delineating the unique molecular features of each subphenotype, we seek to deepen our understanding of inflammatory heterogeneity in AP, identify key therapeutic targets, and advance precision fluid management strategies for AP.^43^

Our study has strengths. It represents the largest analysis to date of the development of AP subphenotypes using a multivariate longitudinal trajectory. Using readily available bedside vital signs, we derived AP subphenotypes in the ICU using data from a multisource database in China and validated the stability and consistency of these findings in the US population, enhancing their clinical utility and applicability. In addition, our study is the first to establish correlations between AP subtypes and clinical treatments. Using machine learning techniques, we explored the heterogeneity in response to fluid resuscitation among different AP subphenotypes, providing a novel approach for precision treatment options in AP clinical management. In terms of limitations, our study focused exclusively on ICU-admitted AP patients, which may limit the generalizability of our findings. However, mild AP cases, associated with lower mortality risk and standard care, are usually self-limiting, requiring only brief hospitalization, while ICU-admitted AP patients often present with severe inflammation, fluid imbalances, and organ failure, leading to higher mortality risk. Consequently, more precise disease classification is necessary for AP ICU inpatients to optimize treatment strategies and support clinical decisions. Additionally, as a retrospective cohort study, our data inherently contained outliers, unrecorded variables, high missing rate variables, and potential confounders that could impact result reliability. To address this, we compiled a large multinational EHR dataset of critical care AP cases, conducted rigorous internal validation and sensitivity analyses to determine optimal subgroups, and validated the stability of AP clinical subtypes in external cohorts. Nevertheless, potential biases that cannot be completely eliminated should still be noted. Furthermore, due to the limitations of the retrospective design, including missing weight data, fluid resuscitation recommendations for different subphenotypes could not be expressed as mL/kg rates. Therefore, individualized fluid intake needs to be further validated in future prospective studies.

In conclusion, our study unveiled four distinct AP phenotypes derived from EHR data, illustrating diverse clinical profiles, prognoses, and responses to fluid replacement in ICU-managed AP patients. External validation across international datasets substantiated the stability of these subtypes, enhancing our insights into AP pathophysiological nuances. Leveraging machine learning, we delved into personalized fluid resuscitation strategies tailored to different AP subphenotypes. These findings warrant further validation through future research endeavors.

## Materials and methods

### Study design and population

**Fig. 5:**
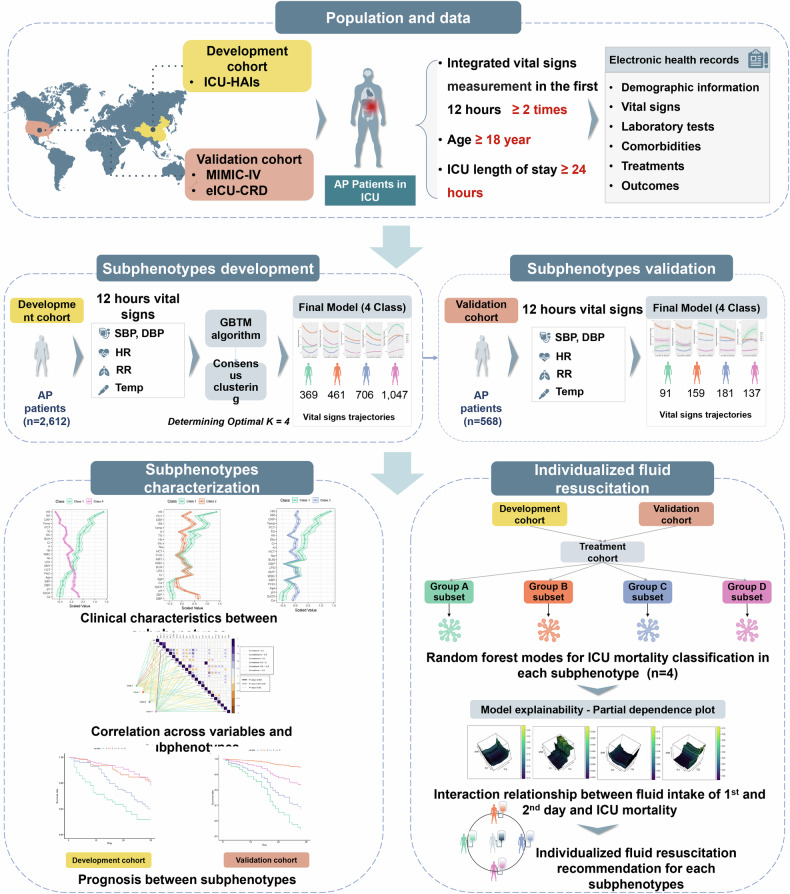
Schema of the study design and progress

To develop dynamic clinical subphenotypes and explore the unique clinical traits across subphenotypes to guide further precision treatment, we conducted a retrospective, multicenter, observational cohort study. The overall workflow chart was exhibited in Fig. 5. Eligibility for the study population encompassed diagnosed AP patients in development and validation cohorts. In cases of multiple ICU admissions, only the first entry was utilized for analysis. Exclusions covered individuals aged under 18, those with ICU length of stay (LOS) shorter than 24 h, and those lacking at least two time points of vital observation within 12 h. We used an extensive repository of electronic health record (EHR) data extracted from three real-world critical care databases. These databases included the ICU healthcare-associated infections (ICU-HAI) database, sourced from the West China Hospital^44,45^; the Medical Information Mart for Intensive Care (MIMIC)-IV originating from the Beth Israel Deaconess Medical Center in the United States^46^; and the eICU Collaborative Research Database (eICU-CRD), a comprehensive multicenter intensive care repository including over 200 hospitals spanning the United States.^47^

After data preprocessing, the ICU-HAI dataset was designated as the development cohort for AP subphenotyping, and the combination of the MIMIC-IV and eICU-CRD datasets served as the external validation cohort. The characteristics and prognosis of patients in different subphenotypes were compared after the identification of AP subphenotypes. Subsequently, the development and validation cohorts containing subphenotype labels were pooled into the treatment cohort to examine the differential associations between fluid resuscitation strategies and mortality across subphenotypes (Fig. 5).

### Model development for subphenotyping

#### Vital sign trajectories data preparation

Vital signs [systolic and diastolic blood pressure (SBP and DBP), heart rate, respiratory rate (RR), and body temperature (TEMP)] measurements for AP patients were aggregated hourly for 12 h after ICU admission. Multiple measurements within the same hour were averaged. Our analysis was restricted to complete hourly datasets, therefore any hour measurement containing a missing value of the five vital signs was omitted to maintain data integrity. Further details on the preprocessing of vital sign trajectories can be found in Supplementary Method A. Prior to fitting the vital sign trajectories into the model, all values of vital signs were z-score standardized to mitigate the impact of variable magnitudes on the model’s fitness.

#### Group-based multi-trajectory model (GBMTM) development

GBMTMs were employed for subtyping AP patients based on their vital signs trajectory during the first 12 h after ICU admission. GBMTM is an unsupervised algorithm that identifies distinct trajectory patterns in multivariate time-series data, using finite mixture models interfaced with polynomial regression, and parameter estimation is performed through the expectation-maximization algorithm to group observations based on their underlying subpopulation distributions. Compared to traditional trajectory analysis methods that often rely on single-variable models, GBMTM can simultaneously analyze multiple markers and cluster based on multiple trajectories, offering a more accurate representation of a patient’s true disease progression. After data preprocessing, the standardized data were incorporated into GBMTMs to delineate distinct AP subphenotypes, which were characterized by unique polynomial regression functions that captured the vital signs’ temporal trajectory^48^ (Supplementary Method B).

#### GBMTM model validation

Internal validation was conducted using the development cohort data to assess subtyping strategies across GBMTMs. Longitudinal consensus clustering algorithms were employed to gauge the stability and reliability of potential subtype numbers (K), ranging from 2 to 6. The evaluation criteria for identifying the optimal K encompassed the elbow point of the delta area under a cumulative distribution function plot with mean consensus scores >0.8 for all subgroups (Supplementary Methods C). For sensitivity analysis, model evaluation encompassed the computation of various metrics and a restricted minimal sample size, including the Akaike information criterion, Bayesian information criterion, integrated completed likelihood criterion, and entropy. To ensure clinical relevance, the size of potential subphenotypes was constrained to a maximum of 10% of the total population.^49^

The final model was applied to the external validation cohort. In the validation cohort, each patient’s individual vital signs data were fitted to the various components (mixture polynomial functions) within the trained GBMTM. The mean squared error (MSE)s were calculated for each component’s fit. Patients were then assigned to the subphenotype corresponding to the component that yielded the lowest MSE.

### Early prediction of subphenotypes before 12 h

To test whether fewer vital sign measurements were sufficient for classification, we evaluated the agreement between the full 12-h model and models with 6 to 12 h of vitals data. Predictions were also conducted based on MSE calculations.

### Clinical characteristics and prognosis between subphenotypes

Clinical characteristics including demographic data (age, gender, race), baseline vital signs (heart rate, RR, SBP, DBP, TEMP, and oxygen saturation), baseline laboratory test (amylase, lipase, blood urea nitrogen (BUN), creatinine, sodium, potassium, calcium (Ca), phosphate, white blood cell count (WBC), C-reactive protein (CRP), procalcitonin (PCT), hemoglobin (Hb), hematocrit, pH, glucose, and triglycerides), were meticulously gathered on the first day of ICU admission. Additionally, comorbidities including myocardial infarction, congestive heart failure, cerebrovascular disease, chronic pulmonary disease, diabetes, liver disease, renal disease, and malignant tumors, were also documented.

Continuous variables were presented as the median (interquartile range), while categorical variables were expressed as counts (percentages). Differences between subphenotypes in continuous variables were assessed using Kruskal–Wallis tests, and *χ*^2^ tests were used for comparing categorical variables.

We compared 30-day mortality between AP subphenotypes using adjusted Kaplan–Meier (KM) plots in the development and validation cohorts. Hazard ratios (HRs) across subphenotypes were estimated through Cox proportional-hazards regression models. KM curves and Cox regression models were adjusted by age, gender, and race.

### Impact of comorbidities on prognoses across subphenotypes

To evaluate the impact of comorbidities on 30-day mortality by subphenotypes, we included those with a prevalence >5% in a multivariate Cox proportional-hazards regression and calculated HRs.

### Heterogeneous treatment response to fluid resuscitation across subphenotype

To investigate heterogeneous responses to fluid resuscitation among distinct subphenotypes, the development and validation cohorts were merged into the treatment cohort encompassing all enrolled patients. The volume of fluid intake during the initial 48 h following ICU admission was measured and summarized for each day. Subsequently, random forest (RF) classifiers were constructed for ICU mortality, tailored to different subphenotype groups. The features of these classification models included common elements present in all datasets, comprising clinical characteristics and fluid volume intake during the initial two days following ICU admission. A fivefold cross-validation was employed for hyperparameter optimization, with the receiver operating characteristic curve serving as the metric to evaluate model accuracy. Finally, multi-predictor interactive partial dependence plot (PDP) algorithms we utilized to probe the relationship between fluid volume intake during the first and second days and the risk of mortality^50^ (Supplementary Method D). Additionally, we examined the effect of fluid choice on prognosis across subphenotypes. Using Cox regression models adjusted for age, gender, and race, we calculated the relative HR for patients receiving lactated Ringer’s solution within 48 h compared to those who did not.

### Missing data management

For the vital sign trajectories, there was no need for missing data processing since any hourly measurement with missing values was omitted. For the clinical characteristics, the missing rates for variables were calculated and reported (Supplementary Table 8). To ensure the accuracy of the results, we excluded variables with missing rates over 20% before conducting univariable analyses of clinical characteristics and fitting the RF model. All variables in the development cohort were included in the descriptive analysis. In the validation cohort, amylase, CRP, PCT, and triglycerides were excluded, while the remaining variables were analyzed. For the remaining missing data, four imputation algorithms (mean, CART, RF, Lasso.norm) were conducted, generating four datasets which were then integrated by calculating their mean values, yielding the final comprehensive dataset.^51^ These variables were subsequently used to construct the RF model in the treatment cohort (Supplementary Method E).

The data from the MIMIC-IV and eICU-CRD (validation cohort) were extracted using structured query language (SQL) in PostgreSQL (version 15). All data were analyzed within the R environment (version 4.3.1), and a list of dependent packages was provided (Supplementary Table 9). For all statistical tests, a two-tailed *P* value of <0.05 was considered statistically significant.

## Supplementary information

Supplementary Materials

## Data Availability

The data from public databases were available on the MIMIC-IV website at https://mimic.physionet.org/, and the eICU-CRD website at 10.13026/C2HM2Q. Other data/materials in this article can be reasonably applied to the corresponding author(sunxin@wchscu.cn).
