# Supplementary material for: Development and validation of dynamic clinical subphenotypes in acute pancreatitis patients using vital sign trajectories in intensive care units: a multinational cohort study
Source: Signal Transduct Target Ther. 2025 Jun 4;10:180. doi: 10.1038/s41392-025-02261-4 (PMC12137743; doi:10.1038/s41392-025-02261-4)
Supplement: Supplementary file 1 — Supplementary Materials [file 41392_2025_2261_MOESM1_ESM.docx]

Supplementary Materials for

Development and validation of dynamic clinical subphenotypes in acute pancreatitis patients using vital sign trajectories in intensive care units: a multinational cohort study

Zichen, Wen Wang, Jiayue Xu, Qiao He, Che Sun, Shuangyi Xie, Kang Zou, Qing Xia, Xin Sun

Correspondence to: Wen Wang (wangwen83@outlook.com) and Xin Sun (sunxin@wchscu.cn

**This PDF file includes:**

Materials and Methods

Figures. S1 to S8

Tables S1 to S9

Materials and Methods

A. Vital sign trajectory preprocessing

Vital sign trajectories of AP patients within the first 12 hours after admission to the ICU are stored as multidimensional longitudinal data. For each patient, systolic blood pressure (SBP), diastolic blood pressure (DBP), heart rate (HR), respiratory rate (RR), and body temperature (TEMP) were hourly averaged and aggregated. This entails calculating the mean of all measurements within a given hour if a vital sign is measured multiple times. This preprocessing ensures that there are no duplicate values in the longitudinal data for each patient. Additionally, records with missing data for any of the five vital signs at the single time point are excluded. A plot illustrating the data processing is presented below:


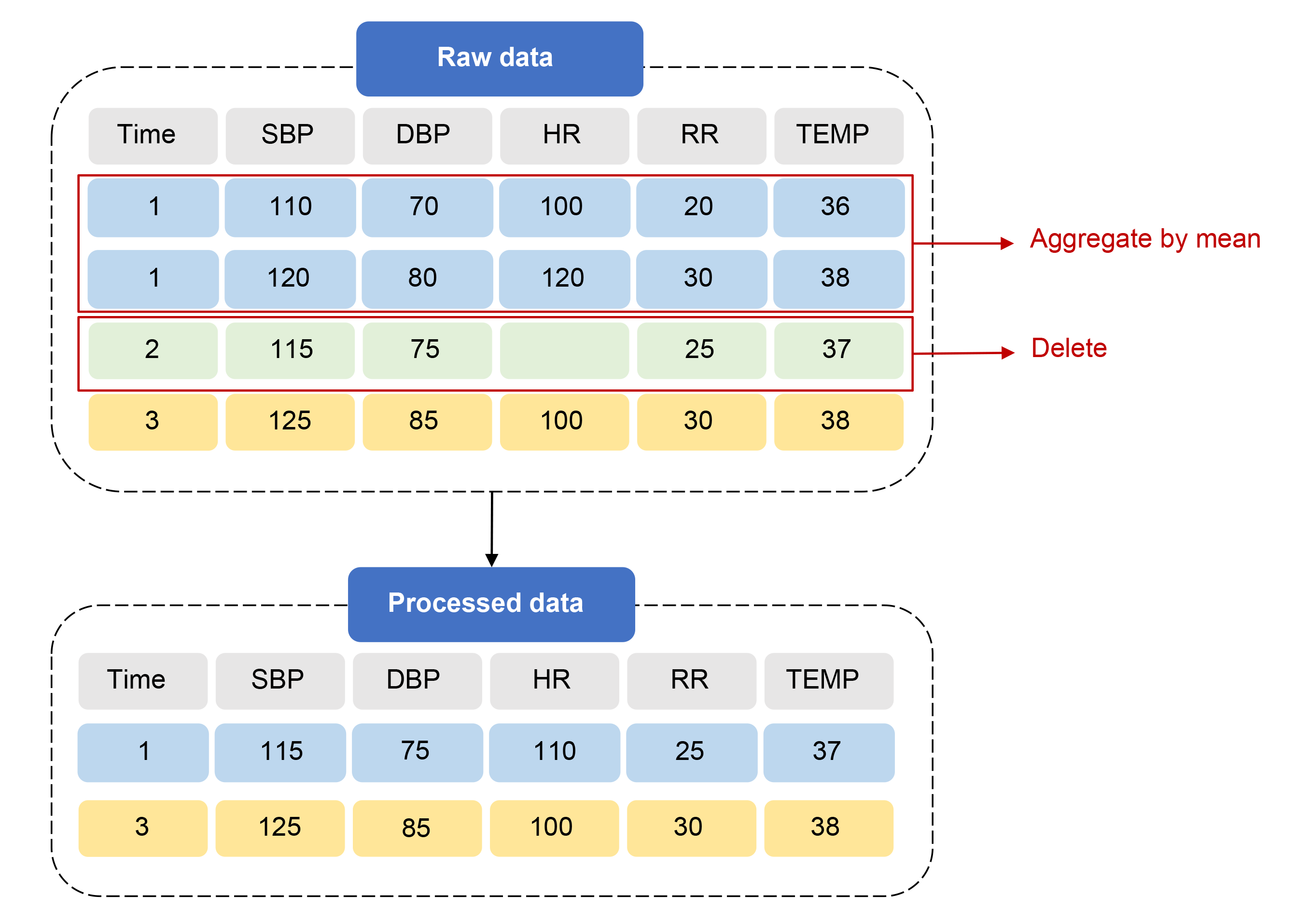


In this plot, each color represents an individual AP patient (blue - Patient A, green - Patient B, yellow - Patient C). Patient A had multiple measurements at time 1, leading to the averaging and aggregation of all vital sign records. Patient B, having records only for time 2 with a missing HR, had the entire row of records deleted. Patient C, with no multiple measurements or missing records, was retained as is. In addition, patients without at least 2 vital sign observations within 12 hours were excluded in the next step to meet the minimum requirements for trajectory composition. Subsequently, all vital sign records were fitted into models for analysis.

B. Group based multivariable trajectory model (GBMTM)s development

GBMTMs were constructed using finite mixture algorithms interfaced with linear regression models. These models assume that the data is generated by a mixture of several underlying subpopulations or components, each with its own probability distribution. In GBMTM, the conditional probability distribution function of each latent class is multivariate, meaning it is a function composed of multiple independent variables. During the parameter estimation process, the Expectation-Maximization algorithm (EM algorithm) is typically employed to estimate model parameters. This iterative algorithm alternates between an E-step, where it estimates the posterior class probabilities for each observation, and an M-step, which maximizes the log-likelihood for each component independently using the posterior probabilities as weights. The EM process continues until either the likelihood improvement falls below a predefined threshold or a maximum number of iterations is reached. Below is a sample R code snippet for constructing a GBMTM:

| Model <- flexmix(~Hours+I(Hours^2)\|ID, data=Vital_Sign, K=K, control=list(inter.max=1000, nrep=10000),  model=list(  FLXMRglm(SBP~. family="gaussian"),  FLXMRglm(DBP~. family="gaussian"),  FLXMRglm(HR~. family="gaussian"),  FLXMRglm(RR~. family="gaussian"),  FLXMRglm(TEMP~. family="gaussian"),  )) |
| --- |

'K' represents the number of potential classes, 'inter.max' stands for the maximum number of iterations, and 'nrep' reports the number of random initializations used in the iterative algorithm to determine the mixture.

SBP: systolic blood pressure; DBP: diastolic blood pressure; HR: heart rate; RR: repatriation rate; TEMP: temperature.

C. Consensus clustering

Consensus clustering (CC) is a resampling-based method used to determine the number and membership of potential clusters within a dataset. By resampling data subsets, CC constructs a consensus matrix, visually displayed to help researchers identify a reasonable number of clusters. Essentially, CC does not create a clustering strategy itself but evaluates existing clustering strategies through resampling and selects the optimal number K. In this study, we employed CC to determine the optimal K in GBMTM.

First, CC randomly selects a certain proportion of sub-populations based on different clustering schemes (K=2-6) and fits them into the GBMTM. For this study, the model was set to extract 80% of the samples each time and iterate 100 times. Each scheme outputs a Consensus Matrix (n=5). The dimensions of each Consensus Matrix are: the number of included patients × the number of included patients. If two samples are always in the same cluster during 100 iterations, their consensus value is 1; if they are never in the same cluster, it is 0. Thus, the Consensus Matrices output in this study are all matrices with dimensions of 2612 × 2612, and each element has a value range of 0-1.

Second, based on the generated Consensus Matrix, we can draw the Cumulative Distribution Function (CDF) plot for the elements in the matrix between 0 and 1 and the Delta Area Plot, which reflects the area under the CDF curve as K changes. The elbow point of the Delta Area Plot is considered the optimal K because it marks where the increase in the number of clusters leads to a significant decrease in consensus, thus balancing model complexity and clustering quality.

Finally, clusters beyond the elbow point may lead to overfitting, while clusters before it better capture the data's structure. Finally, clear consensus matrix heatmap boundaries and the mean consensus scores (the mean of all consensus matrix elements in a subgroup) greater than 0.8 were used to evaluate the stability of the grouping strategy for each specific subgroup and support identifying the final optimal K.

D. Random forest classifier based partial dependence plots

To explore the specific response of each subphenptype to fluid resuscitation, we used partial dependence plots (PDP) based on the random forest (RF) model. This method mainly consists of two parts: 1. training of the RF model and 2. deconstruction of the trained RF by PDP.

**Random forest classifier training**

All patients from the development and validation cohorts were merged into a single treatment cohort containing features including *age, gender, race, systolic blood pressure, diastolic blood pressure, heart rate, repatriation rate, temperature, oxygen saturation, lipase, blood urine nitrogen, creatinine, sodium, potassium, calcium, phosphate, white blood cell, hemoglobin, hematocrit, pH, glucose, fluid intake 1st day, fluid intake 2nd day, ICU length of stay, hospital length of stay, myocardial infarction, congestive heart failure, cerebrovascular disease, chronic pulmonary disease, diabetes, hepatobiliary disease, renal disease, malignant tumor, and ICU mortality.*

For each dataset, we constructed a RF classifier to distinguish ICU mortality. We employed a five-fold cross-validation for parameter optimization (mtry) for each classifier . To assess model performance, we utilized Receiver Operating Characteristic (ROC) curves and Area Under the ROC curve (AUROC) to ensure that each model effectively captures the risk of ICU mortality. The final classifier for each subphenotype will be integrated into subsequent analyses. Parameters of final classifiers were showed:

| Model | RF_A | RF_B | RF_C | RF_D |
| --- | --- | --- | --- | --- |
| **Hyperparameters** |  |  |  |  |
| mtry | 7 | 1 | 7 | 19 |
| ntree | 500 | 500 | 500 | 500 |
| **Gini importance** |  |  |  |  |
| Age | 3.291 | 0.608 | 4.103 | 1.643 |
| Gender | 0.431 | 0.191 | 0.811 | 0.082 |
| Race-Black | 0.000 | 0.013 | 0.161 | 0.000 |
| Race-Asia | 0.145 | 0.133 | 0.199 | 0.011 |
| Race-Others | 0.086 | 0.020 | 0.217 | 0.009 |
| Systolic Blood Pressure | 2.921 | 1.021 | 4.921 | 0.805 |
| Diastolic Blood Pressure | 4.795 | 0.985 | 4.330 | 1.052 |
| Heart Rate | 3.681 | 0.728 | 4.091 | 1.596 |
| Repatriation Rate | 3.262 | 0.579 | 2.939 | 1.642 |
| Temperature | 4.128 | 0.755 | 3.023 | 1.611 |
| Oxygen Saturation | 3.315 | 1.037 | 8.498 | 7.275 |
| Amylase | 4.480 | 0.954 | 5.867 | 1.132 |
| Lipase | 4.550 | 0.907 | 4.837 | 0.980 |
| Blood Urine Nitrogen | 2.490 | 1.200 | 5.968 | 1.671 |
| Creatinine | 5.208 | 1.392 | 4.995 | 4.089 |
| Sodium | 4.513 | 1.001 | 5.854 | 4.026 |
| Potassium | 3.282 | 0.878 | 4.143 | 0.967 |
| Calcium | 4.245 | 0.900 | 3.665 | 1.464 |
| Phosphate | 3.310 | 0.734 | 4.203 | 2.419 |
| White Blood Cell | 3.578 | 0.620 | 6.491 | 2.695 |
| C-Reactive Protein | 2.544 | 0.734 | 4.823 | 1.810 |
| Hemoglobin | 3.192 | 0.776 | 4.155 | 2.351 |
| Hematocrit | 3.265 | 1.003 | 3.373 | 1.892 |
| pH | 8.211 | 1.107 | 9.229 | 1.136 |
| Glucose | 3.533 | 0.799 | 4.292 | 1.164 |
| Triglycerides | 2.615 | 0.848 | 3.932 | 1.759 |
| Hospital Length of Stay | 2.116 | 0.925 | 2.642 | 3.864 |
| ICU Length of Stay | 2.150 | 0.693 | 2.426 | 0.876 |
| Myocardial Infarction | 0.005 | 0.004 | 0.350 | 0.017 |
| Congestive Heart Failure | 0.274 | 0.117 | 0.367 | 0.375 |
| Cerebrovascular Disease | 0.375 | 0.141 | 1.665 | 0.775 |
| Chronic Pulmonary Disease | 0.311 | 0.007 | 0.340 | 0.170 |
| Diabetes | 0.370 | 0.181 | 0.573 | 0.121 |
| Hepatobiliary disease | 0.440 | 0.166 | 0.867 | 0.164 |
| Renal Disease | 0.538 | 0.126 | 0.948 | 0.171 |
| Malignant Tumor | 0.100 | 0.021 | 0.353 | 0.066 |

**PDPs generating**

PDP is model explanation and interpretation algorithms that reveal how the probability of a target outcome changes as the value of one or more features increases. PDPs can capture the non-linear 'dose-response' relationship between specific features and outcomes by calculating the average effect of other predictors. In this study, PDPs were applied to the previous trained RF classifiers. Next, the PDPs will generated a data frame contained probability of ICU mortality under combination of fluid intake 1st day and fluid intake 2nd day. Below is a sample R code snippet for constructing a PDPs results:

| PDP <- partial(RF_Classifier, pred.var=c(‘Fluid intake 1st day’, ‘Fluid intake 2nd day’, which.class= ‘ICU Mortality’) |
| --- |

RF_Classifier: The trained Classifier for Phenotype A-D;

pred.var: Names of the predictor variables of interest;

which.class: Specifying which column of the matrix of predicted probabilities to use as the "focus" class.

E. Missing data and imputation

Error records and outliers in the original data were meticulously identified and removed through consultations and consensus among clinical experts. Subsequently, variables with a missing proportion exceeding 20% were excluded from the development cohort (None) and the validation cohort (Amylase, C-Reactive Protein, Procalcitonin, Triglycerides), respectively. We implemented a more stringent missing data tolerance threshold to ensure the provision of precise and robust information for subsequent model development within the combined treatment cohort. Only features shared between the development and validation cohorts were retained in the treatment cohort. We assumed that all data were missing conditionally dependent on other variables that were not missing in each sample, thus satisfying the criteria for multiple imputation by chained equations (MICE). Therefore, an imputation framework based on MICE was performed independently on each dataset. Four data imputation techniques were employed to generate four distinct datasets, including unconditional mean imputation (mean), classification and regression tree (CART), random forest imputations (RF), and lasso linear regression (Lasso.norm). Finally, the complete datasets generated by different algorithms were merged using means, resulting in a unified complete dataset. As shown in the figure below:


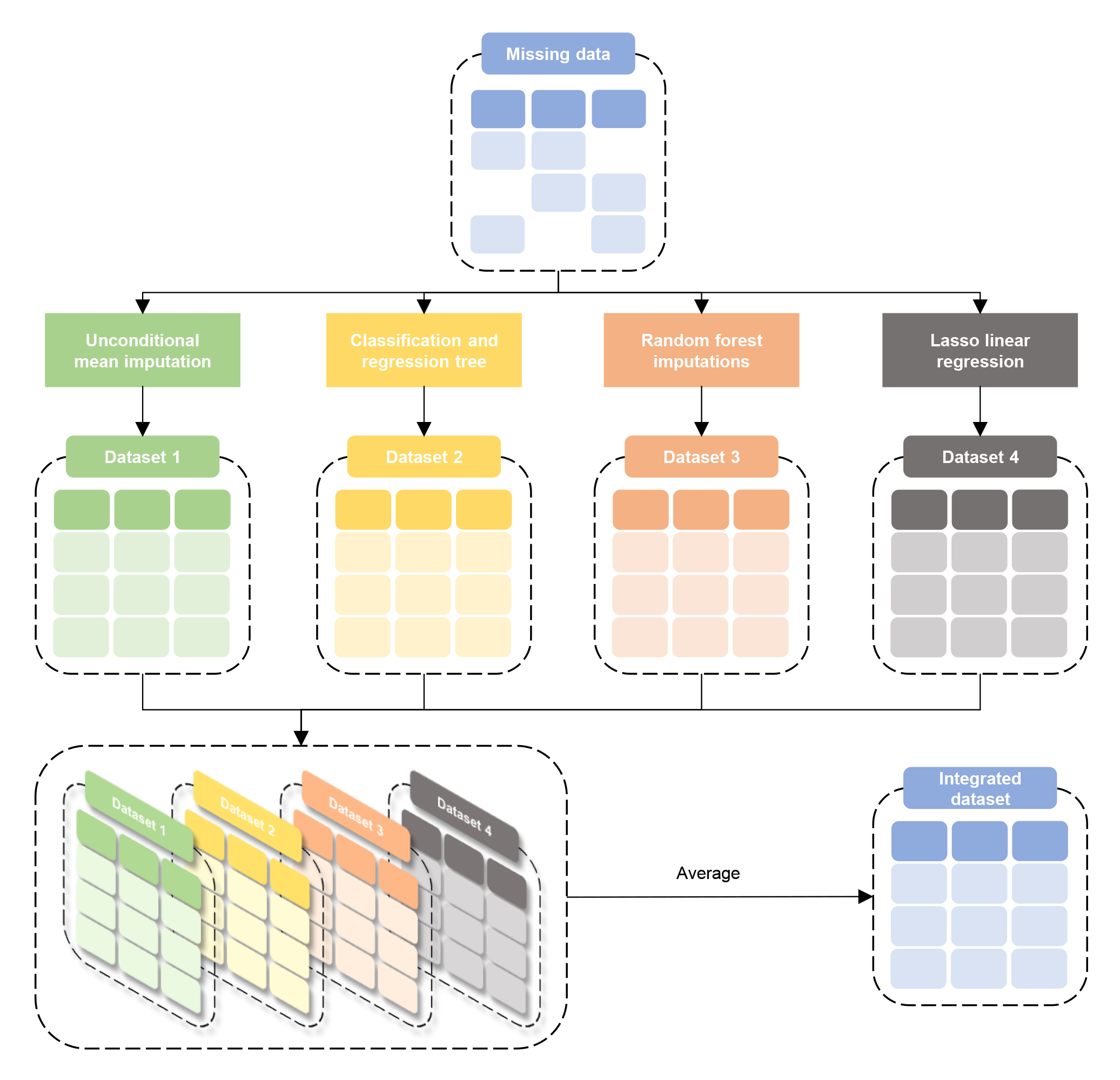


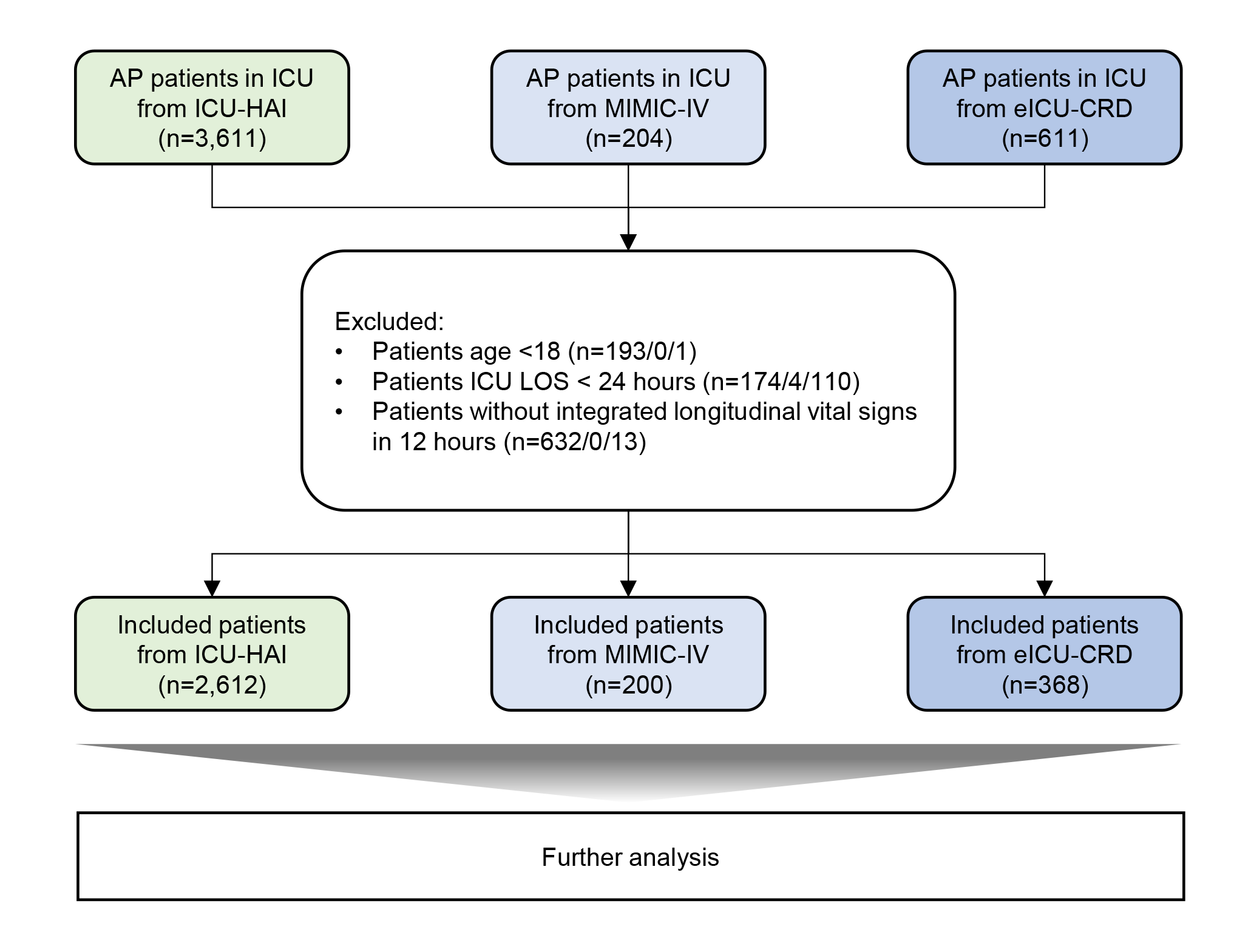


Figure. S1. Inclusion exclusion flow chart for the study


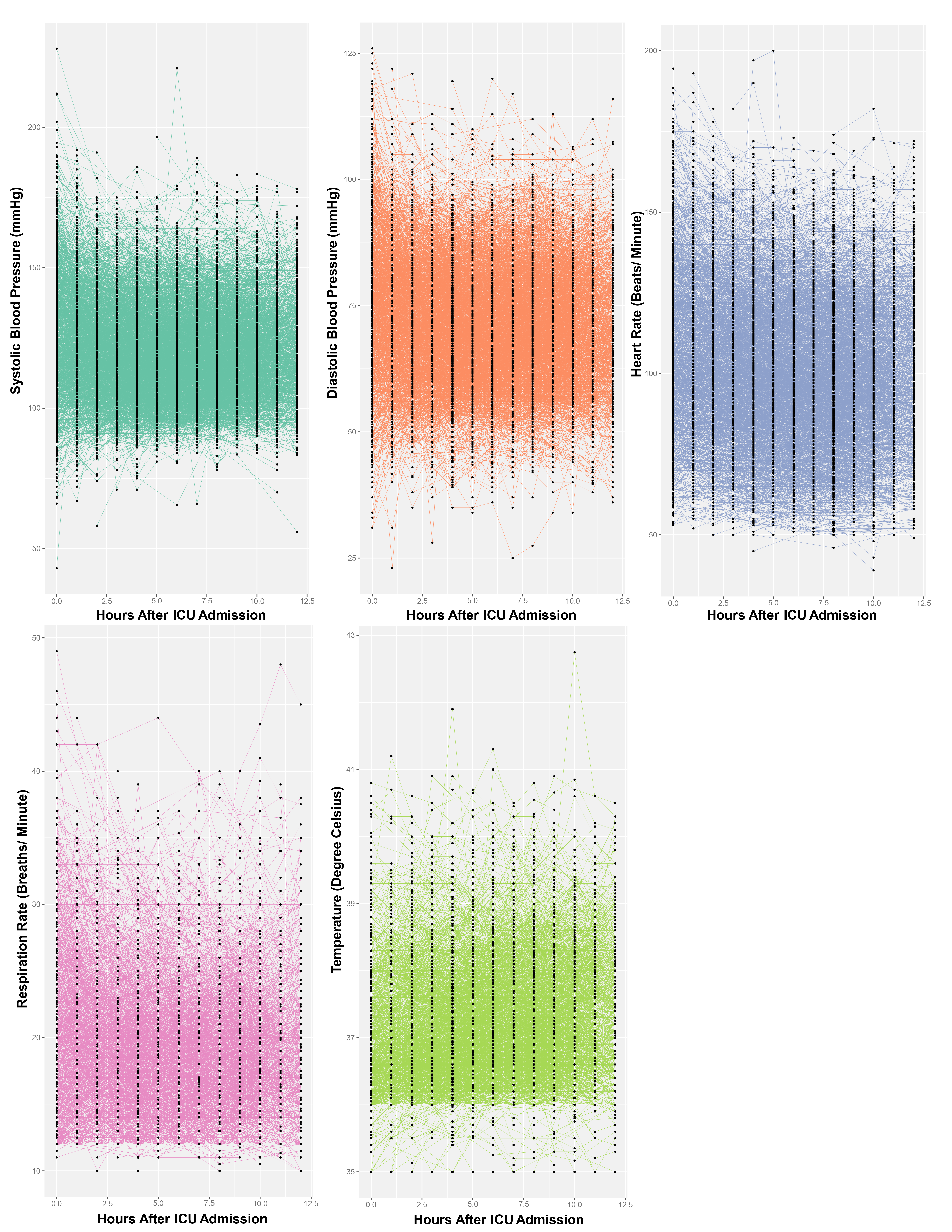


Figure. S2. Trajectories of vital signs of AP patents in the first 12 hours after ICU admission among development cohort The trajectories of vital signs, including systolic blood pressure, diastolic blood pressure, heart rate, respiratory rate, and body temperature, for patients with AP within the initial 12 hours of ICU admission. Each data point corresponds to a measurement, and each line represents the individual trajectory of a patient.

Type or paste caption here. Create a page break and paste in the Figure above the caption.


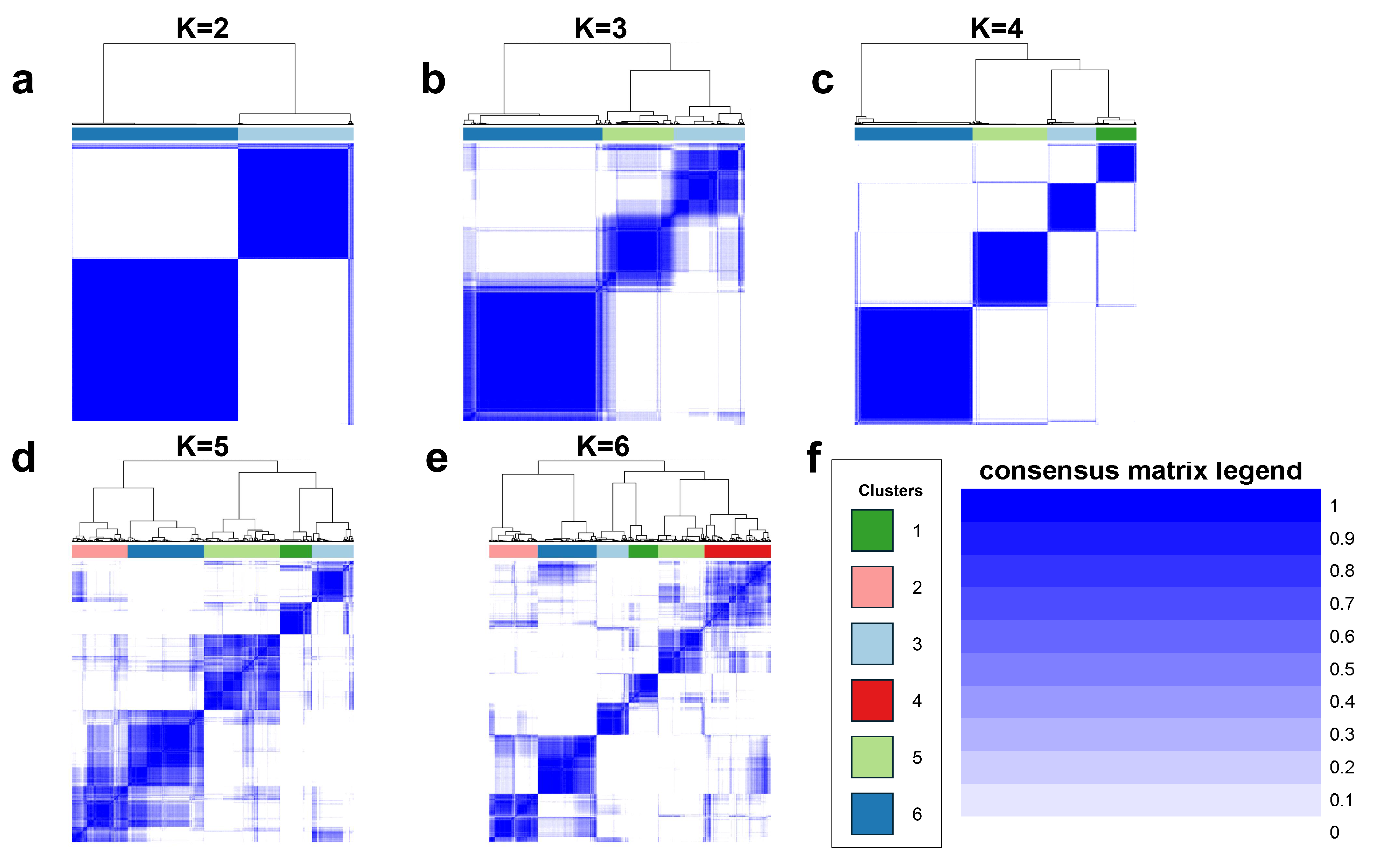


Figure. S3. Consensus matrices (K= 2-6) The consensus matrices for AP subphenotypes (K=2-6) were presented in the form of microarrays(Supplementary Fig. 3a-e). These matrices capture consensus values ranging from 0 (indicating instances where subphenotypes were never clustered together) to 1 (indicating instances where subphenotypes were always clustered together). The visualization of these matrices was represented as heatmaps, with colors ranging from white (value=0) to dark blue (value=1) (Supplementary Fig. 3f). The arrangement of the consensus matrices followed the order of consensus clustering, which is visually depicted as a dendrogram positioned above the heatmap. Notably, when K=2 and 4, distinct groups within the heatmap exhibited well-defined boundaries, which signifies the robustness and stability of the identified subgroups. These clear boundaries underscore the consistency in the clustering results, where subphenotypes within the same group consistently clustered together across multiple iterations, as indicated by the high consensus values in the corresponding cells of the heatmap.


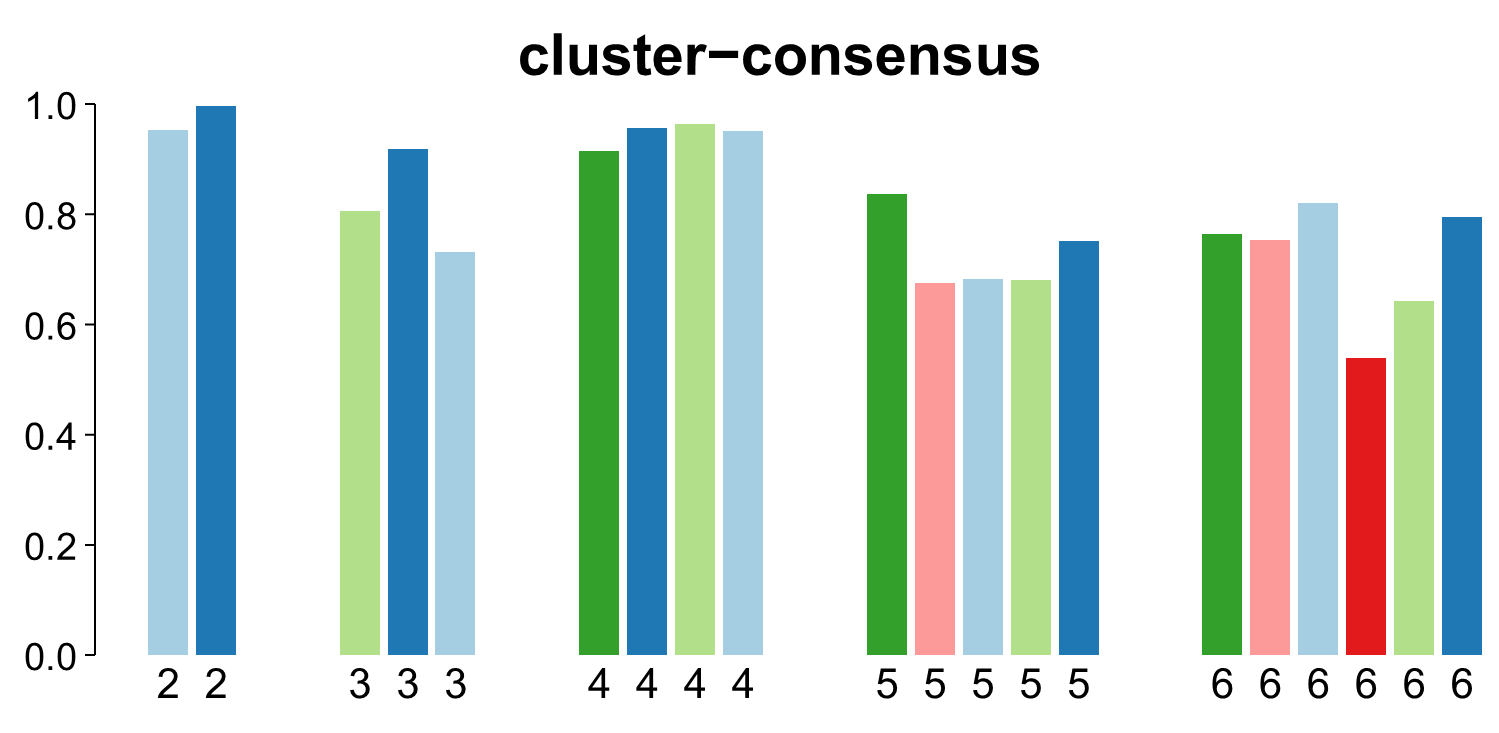


Figure. S4. Cluster-consensus plot The Cluster-consensus plot reveals the cluster-consensus values for clusters at each value of K, ranging from K=2 to 6. These values are computed by taking the mean of all pairwise consensus values between the members of a cluster. A consensus value exceeding 0.9 is generally indicative of strong cluster stability. As depicted in the figure, when K=2 and K=4, the consensus values for different subgroups consistently exceed the 0.9 threshold. This pattern of high consensus values at K=2 and K=4 underscores the robustness and stability of the identified clusters. It suggests that these particular values of K lead to well-defined and internally cohesive subgroups, as evidenced by the strong consensus among their members. These findings support the selection of K=2 and K=4 as optimal choices for capturing meaningful subphenotypes within the dataset.


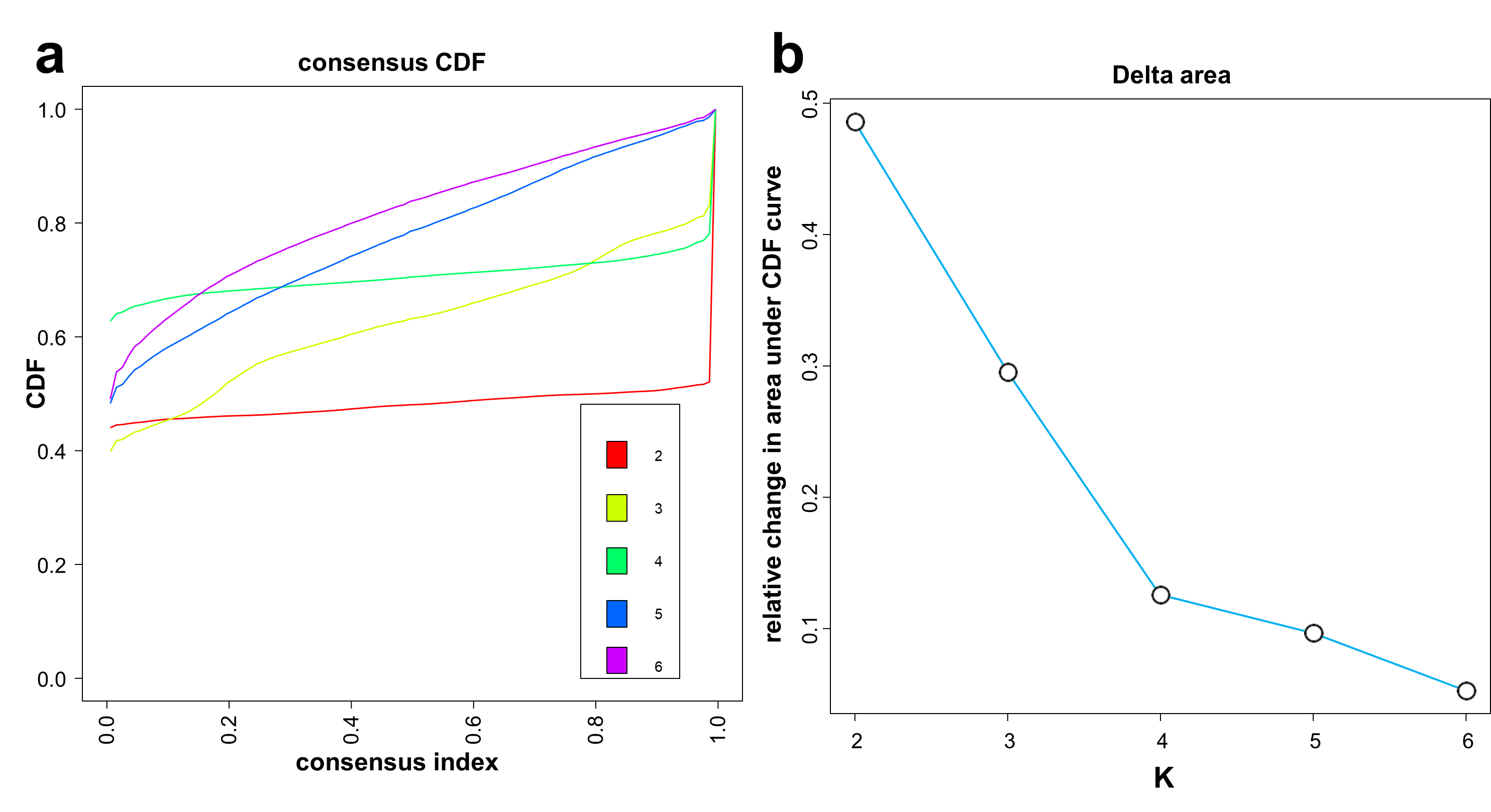


Figure. S5. Consensus cumulative distribution function (CDF) plot and delta area plot The CDF plot, as shown in Supplementary Fig. 5a, illustrates the consensus distributions across different values of K, ranging from K=2 to 6. When comparing these curves, it becomes evident that K=2 and K=4 exhibit the most desirable shape, characterized by the steepest ascent of values in the range between 0 and 1. This steep climb in the CDF curve signifies a strong consensus among the subphenotypes within these clusters. The delta area curve, represented in Supplementary Fig. 5b, quantifies the relative change in area beneath the CDF curve when moving from K to K-1. Notably, the delta area curve highlights K=4 as a distinct elbow point. In simpler terms, K=4 stands out as the point where the relative area change under the CDF curve experiences a significant decrease compared to K=5. This finding indicates that K=4 provides a meaningful level of cluster stability and structure, making it a valuable choice for identifying subphenotypes within the dataset.


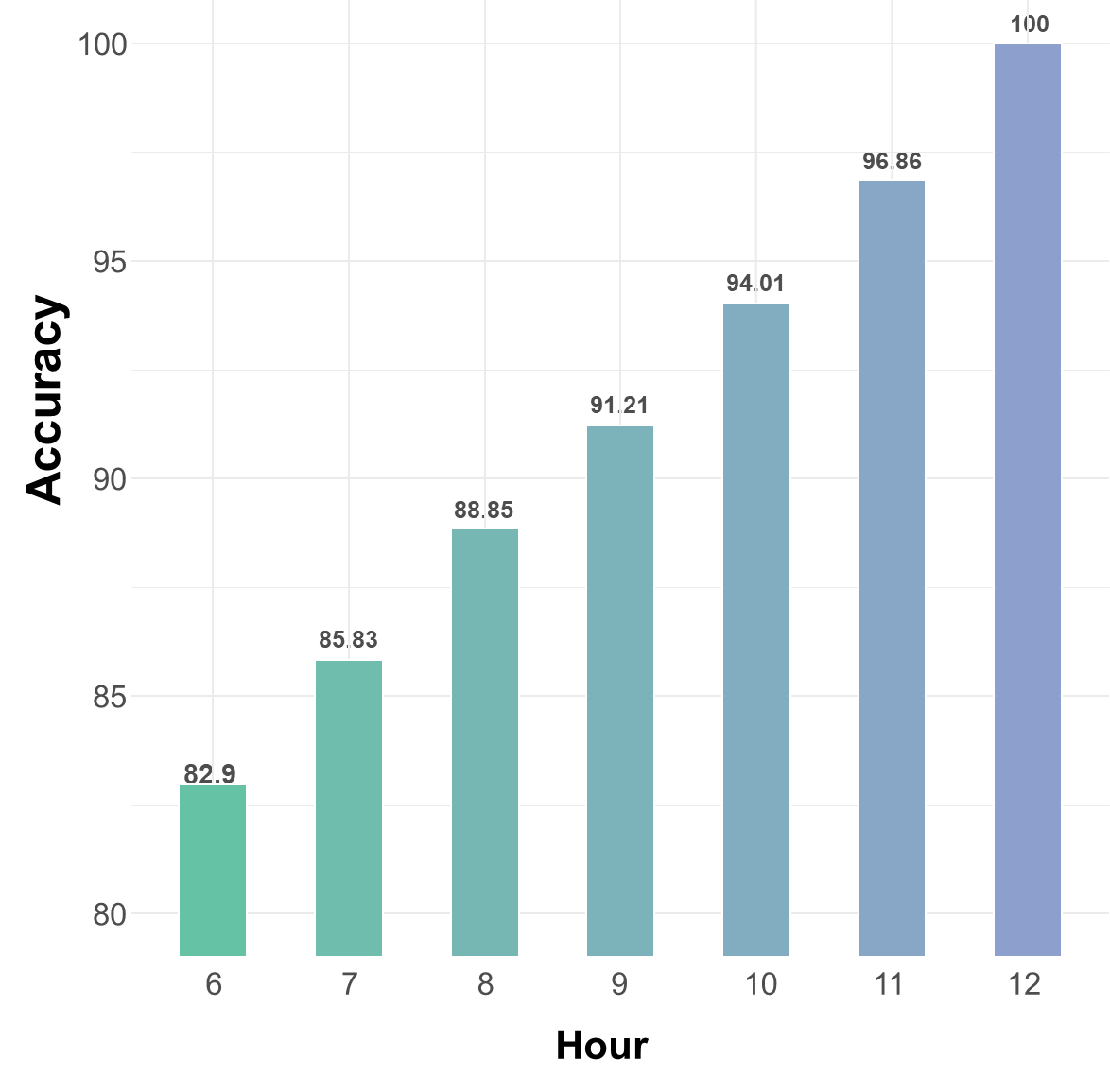


Figure. S6. Prediction accuracy as observation period increasing The accuracy of patient subphenotyping prediction improves from 82.97% (using 0-6 hour data) to 96.86% (using 0-11 hour data) as the trajectory data becomes more complete. With 0-9 hours of data, the model accurately predicts 91.21% of patient phenotypes, an accuracy we find acceptable as it mitigates the impact of treatments on patient trajectories. Thus, clinicians can use the method described in question 2 to predict patient phenotypes before treatment and tailor their interventions accordingly.


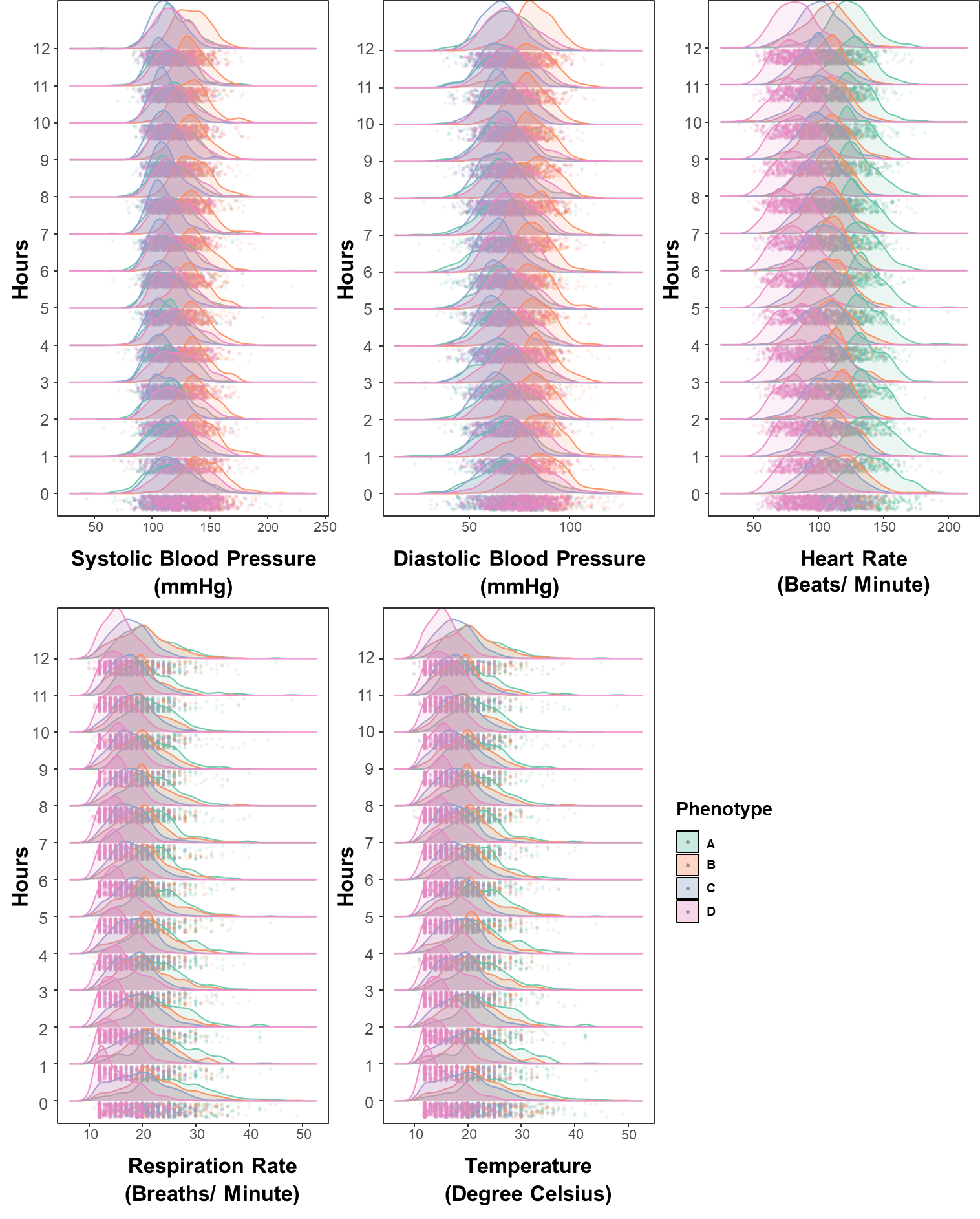


Figure. S7. Distribution of vital signs of AP patents in the first 12 hours after ICU admission among development cohort From left to right is the distribution of SBP, DBP, HR, RR and TEMP based on the phenotypes at 0-12 hours. Patients with phenotype A were obviously higher than those with other phenotypes, with HR showing the greatest difference. Conversely, the distribution of HR, RR and TEMP in phenotype D was significantly lower within the first 12 hours. Regarding blood pressure, both SBP and DBP were significantly higher in phenotype B compared to other phenotypes, and the blood pressure distribution was lower in phenotype C.


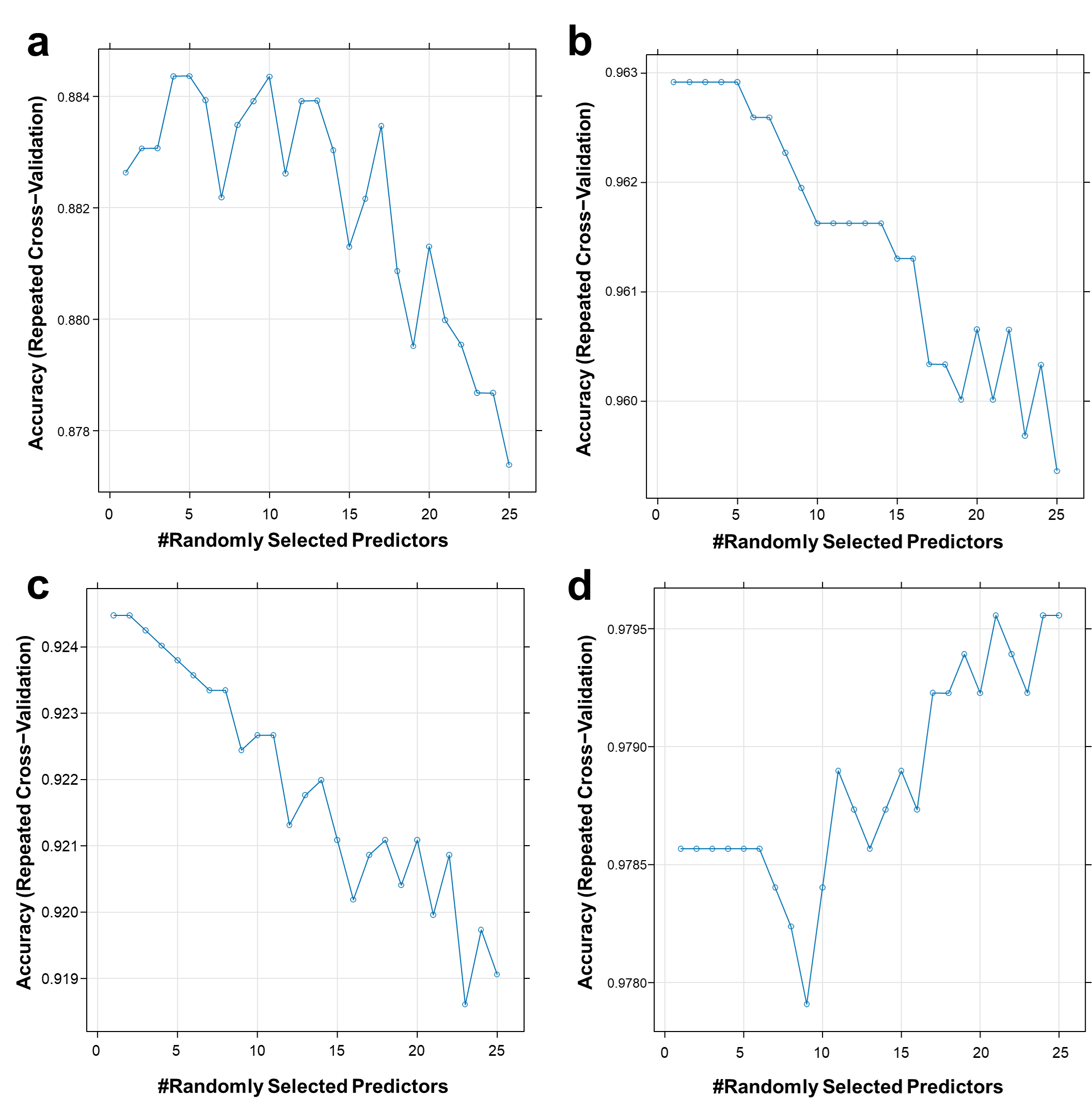


Figure. S8. Process of hyperparameters tunning of random forest classifiers A five-fold cross-validation was performed to fine-tune the hyperparameters of random forest classifiers. Accuracy was employed as the performance evaluation metric across various values of "mtry," which represents the number of variables included in each decision tree.

Table S1. The complete clinical characteristics of subphenotypes in development cohort

| Development cohort (ICU-HAIs registry) | | | | | |
| --- | --- | --- | --- | --- | --- |
| Phenotype | A | B | C | D | p |
| N | 369 | 461 | 706 | 1,076 |  |
| Age | 45.0 (35.0, 53.0) | 45.0 (36.0, 52.0) | 48.0 (39.0, 57.0) | 49.0 (41.0, 60.0) | <0.001 |
| Gender |  |  |  |  |  |
| Male | 235 (63.7) | 329 (71.4) | 457 (64.7) | 720 (66.9) | 0.064 |
| Female | 134 (36.3) | 132 (28.6) | 249 (35.3) | 356 (33.1) |  |
| Race |  |  |  |  |  |
| Asia | 369 (100.0) | 461 (100.0) | 706 (100.0) | 1,076 (100.0) | / |
| Systolic Blood Pressure (mmHg) | 117.0 (102.0, 134.0) | 139.0 (128.0, 154.0) | 116.0 (105.0, 129.0) | 127.5 (112.0, 144.0) | <0.001 |
| Diastolic Blood Pressure (mmHg) | 70.0 (60.5, 80.0) | 85.0 (77.0, 94.0) | 69.5(60.5, 78.0) | 77.5 (69.0, 87.0) | <0.001 |
| Heart Rate (per minute) | 137.0 (125.3, 151.0) | 113.0 (100.0, 126.0) | 106.5 (95.0, 118.5) | 86.0 (75.0, 98.5) | <0.001 |
| Repatriation Rate (per minute) | 24.0 (20.0, 29.0) | 20.5 (16.0, 25.0) | 17.5 (12.5, 21.5) | 12.5 (12.0, 16.0) | <0.001 |
| Temperature (C) | 37.4 (36.6, 38.4) | 37.0 (36.5, 37.6) | 36.8 (36.4, 37.5) | 36.5 (36.2, 36.6) | <0.001 |
| Oxygen Saturation (%) | 96.8 (91.7, 98.8) | 97.6 (95.1, 99.1） | 98.3 (95.9, 99.4) | 99.2(97.8, 99.7) | <0.001 |
| Amylase (U/L) | 207.0 (48.0, 731.5) | 109.0 (42.0, 450.0) | 69.0 (27.0, 339.5) | 77.0 (21.0, 516.9) | <0.001 |
| Lipase (U/L) | 199.0 (38.0, 826.0) | 101.0 (34.0, 495.0) | 55.0 (21.0, 274.8) | 47.0 (9.0, 497.3) | <0.001 |
| Blood Urine Nitrogen (mmol/L) | 9.1 (6.0, 14.0) | 7.2 (4.5, 13.1) | 6.2 (4.0, 11.3) | 4.3 (3.1, 6.2) | <0.001 |
| Creatinine (umol/L) | 109.0 (60.5, 204.7) | 82.0 (54.0, 190.0) | 65.0 (45.0, 120.8) | 58.0 (45.0, 75.6) | <0.001 |
| Sodium (mmol/L) | 138.5 (135.2, 142.3) | 137.3 (134.2, 141.3) | 136.7 (133.6, 141.0) | 136.8 (134.6, 139.2) | <0.001 |
| Potassium (mmol/L) | 4.2 (3.8, 4.7) | 4.0 (3.7, 4.3) | 4.0 (3.7, 4.4) | 4.0 (3.7, 4.3) | <0.001 |
| Calcium (mmol/L) | 1.9 (1.7, 2.0) | 1.9 (1.8, 2.1) | 1.9 (1.8, 2.0) | 2.0 (1.9, 2.2) | <0.001 |
| Phosphate (mmol/L) | 0.9 (0.6, 1.2) | 0.9 (0.6, 1.2) | 1.0 (0.7, 1.3) | 1.1 (0.9, 1.3) | <0.001 |
| White Blood Cell (K/uL) | 12.4 (9.0, 17.0) | 13.0 (8.9, 16.3) | 12.4 (8.4, 18.0) | 10.5 (7.1, 15.0) | <0.001 |
| C-Reactive Protein (mg/L) | 196.0 (118.0, 285.0) | 138.0 (82.8, 213.0) | 124.4 (78.9, 188.8) | 62.6 (19.6, 116.6) | <0.001 |
| Procalcitonin (ng/mL) | 4.4 (1.2, 17.3) | 1.5 (0.5, 5.3) | 1.2 (0.4, 4.3) | 0.3 (0.1, 1.3) | <0.001 |
| Hemoglobin (g/L) | 97.0 (81.0, 129.8) | 96.0 (80.5, 113.0) | 88.0 (76.6, 102.0) | 98.0 (84.0, 114.0) | <0.001 |
| Hematocrit (%) | 30.8 (25.0, 38.7) | 31.2 (26.0, 35.6) | 28.3 (24.5, 33.0) | 31.1 (27.0, 35.5) | <0.001 |
| pH | 7.4 (7.3, 7.4) | 7.4 (7.4, 7.4) | 7.4 (7.4, 7.4) | 7.4 (7.4, 7.4) | <0.001 |
| Glucose (mmol/L) | 10.3 (8.3, 13.0) | 9.7 (7.7, 11.9) | 9.5 (7.5, 11.9) | 8.7 (6.9, 10.8） | <0.001 |
| Triglycerides (mmol/L) | 2.3 (1.3, 5.0) | 2.1 (1.4, 3.4) | 1.6 (1.1, 2.6) | 1.3 (1.0, 1.9) | <0.001 |
| Hospital Length of Stay (Day) | 25.0 (14.0, 39.0) | 25.0 (16.0, 37.0) | 23.0 (14.0, 37.0) | 18.0 (12.0, 27.0) | <0.001 |
| ICU Length of Stay (Day) | 10.0 (5.00, 20.0) | 9.0 (4.0, 17.0) | 7.0 (4.0, 14.0) | 3.0 (2.0, 5.0) | <0.001 |
| Myocardial Infarction (%) | 0 (0.0) | 1 (0.2) | 2 (0.3) | 3 (0.3) | 0.788 |
| Congestive Heart Failure (%) | 33 (8.9) | 28 (6.1) | 57 (8.1) | 53 (4.9) | 0.012 |
| Cerebrovascular Disease (%) | 14 (3.8) | 16 (3.5) | 24 (3.4) | 31 (2.9) | 0.817 |
| Chronic Pulmonary Disease (%) | 11 (3.0) | 17 (3.7) | 22 (3.1) | 72 (6.7) | 0.001 |
| Diabetes (%) | 85 (23.0) | 100 (21.7) | 149 (21.1) | 218 (20.3) | 0.710 |
| Hepatobiliary disease (%) | 143 (38.8) | 180 (39.0) | 254 (36.0) | 489 (45.4) | 0.001 |
| Renal Disease (%) | 41 (11.1) | 36 (7.8) | 66 (9.3) | 54 (5.0) | <0.001 |
| Malignant Tumor (%) | 12 (3.3) | 14 (3.0) | 24 (3.4) | 146 (13.6) | <0.001 |
| ICU Mortality (%) | 46 (12.5) | 23 (5.0) | 47 (6.7) | 22 (2.0) | <0.001 |
| Hospital Mortality (%) | 46 (12.5) | 24 (5.2) | 51 (7.2) | 27 (2.5) | <0.001 |

Table S2. The complete clinical characteristics of subphenotypes in validation cohort

| Validation cohort (MIMICIV, eICU-CRD) | | | | | |
| --- | --- | --- | --- | --- | --- |
| Phenotype | A | B | C | D | p |
| N | 91 | 159 | 181 | 137 | / |
| Age | 45.0 (34.5, 55.5) | 47.0 (35.0, 56.0) | 56.0 (46.0, 70.0) | 60.0 (49.0, 77.0) |  |
| Gender (%) |  |  |  |  | 0.106 |
| Male | 58 (63.7) | 109 (68.6) | 102 (56.4) | 80 (58.4) |  |
| Female | 33 (36.3) | 50 (31.4) | 79 (43.6) | 57 (41.6) |  |
| Race |  |  |  |  | 0.542 |
| White | 66 (72.5) | 105 (66.0) | 126 (69.6) | 102 (74.5) |  |
| Black | 6 (6.6) | 19 (11.9) | 16 (8.8) | 11 (8.0) |  |
| Asia | 0 (0.0) | 2 (1.3) | 5 (2.8) | 1 (0.7) |  |
| Others | 19 (20.9) | 33 (20.8) | 34 (18.8) | 23 (16.8) |  |
| Systolic Blood Pressure (mmHg) | 117.5 (103.2, 133.0) | 144.5 (132.8, 159.0) | 112.0 (101.7, 122.0) | 124.0 (108.0, 143.0) | <0.001 |
| Diastolic Blood Pressure (mmHg) | 69.0 (59.7, 76.7) | 87.3 (78.5, 95.3) | 64.6 (57.7, 71.7) | 69.0 (60.3, 83.0) | <0.001 |
| Heart Rate (per minute) | 123.3 (117.2, 132.2) | 106.6 (97.3, 118.0) | 97.1 (89.0, 107.6) | 79.0 (69.7, 88.8) | <0.001 |
| Repatriation Rate (per minute) | 27.2 (23.5, 32.2) | 22.3 (18.2, 25.5) | 20.9 (18.5, 24.3) | 17.5 (15.0, 20.0) | <0.001 |
| Temperature (C) | 37.3 (36.7, 38.0) | 37.1 (36.8, 37.3) | 37.1 (36.7, 37.4) | 36.7 (36.5, 36.9) | <0.001 |
| Oxygen Saturation (%) | 95.5 (93.8, 97.6) | 96.0 (94.0, 97.5) | 96.0 (94.0, 98.0) | 97.0 (94.8, 98.5) | 0.007 |
| Amylase (U/L) | 209.0 (48.0, 507.9) | 223.0 (74.6, 438.9) | 171.8 (55.0, 339.5) | 193.4 (59.1, 451.5) | 0.221 |
| Lipase (U/L) | 538.0 (107.0, 1430.0) | 426.9 (112.5, 1416.0) | 265.3 (65.0, 915.0) | 418.0 (127.0, 1280.7) | 0.056 |
| Blood Urine Nitrogen (mmol/L) | 8.2 (4.8, 12.0) | 4.6 (2.5, 7.5) | 6.4 (4.3, 11.8) | 6.8 (3.6, 11.4) | <0.001 |
| Creatinine (umol/L) | 113.2 (76.1, 166.7) | 70.7 (53.5, 106.1) | 97.3 (61.9, 176.8) | 97.3 (63.7, 174.2) | <0.001 |
| Sodium (mmol/L) | 139.0 (136.0, 142.0) | 137.0 (135.0, 139.6) | 138.9 (136.0, 141.2) | 138.0 (135.0, 140.2) | 0.006 |
| Potassium (mmol/L) | 1.0 (0.8, 1.2) | 0.9 (0.7, 1.1) | 1.0 (0.8, 1.3) | 1.0 (0.8, 1.2) | <0.001 |
| Calcium (mmol/L) | 1.8 (1.6, 2.0) | 2.0 (1.8, 2.1) | 1.9 (1.8, 2.1) | 2.1 (1.9, 2.2) | <0.001 |
| Phosphate (mmol/L) | 1.0 (0.8, 1.2) | 0.9 (0.7, 1.1) | 1.0 (0.8, 1.3) | 1.0 (0.8, 1.2) | <0.001 |
| White Blood Cell (K/uL) | 12.8 (9.4, 16.6) | 11.4 (8.4, 14.1) | 12.3 (8.9, 17.2) | 9.8 (6.9, 13.9) | <0.001 |
| C-Reactive Protein (mg/L) | 206.6 (46.5, 623.2) | 131.4 (46.1, 379.5) | 171.8 (24.9, 427.4) | 153.8 (17.4, 430.8) | 0.727 |
| Hemoglobin (g/L) | 118.0 (96.0, 137.0) | 128.0 (112.9, 142.0) | 110.0 (95.0, 126.0) | 111.0 (98.0, 127.0) | <0.001 |
| Hematocrit (%) | 36.3 (28.5, 41.0) | 38.1 (33.7, 41.7) | 33.5 (29.0, 38.2) | 33.3 (29.9, 37.4) | <0.001 |
| pH | 7.4 (7.3, 7.4) | 7.4 (7.3, 7.4) | 7.4 (7.3, 7.4) | 7.4 (7.3, 7.4) | <0.001 |
| Glucose (mmol/L) | 8.8 (7.2, 12.4) | 8.4 (6.8, 11.2) | 7.6 (5.7, 11.0) | 7.0 (5.7, 9.1) | <0.001 |
| Triglycerides (mmol/L) | 4.2 (1.9, 8.2) | 4.1 (1.9, 9.1) | 2.7 (1.3, 5.3) | 3.0 (1.1, 6.3) | 0.001 |
| Hospital Length of Stay (Day) | 14.5 (6.9, 22.6) | 5.4 (2.8, 12.1) | 7.2 (3.7, 15.3) | 3.9 (1.4, 9.0) | <0.001 |
| ICU Length of Stay (Day) | 6.3 (3.3, 13.6) | 4.4 (2.5, 9.0) | 6.1 (2.6, 15.2) | 3.9 (2.0, 7.0) | <0.001 |
| Myocardial Infarction (%) | 2 (2.2) | 4 (2.5) | 11 (6.1) | 15 (10.9) | 0.006 |
| Congestive Heart Failure (%) | 10 (11.0) | 5 (3.1) | 29 (16.0) | 20 (14.6) | 0.001 |
| Cerebrovascular Disease (%) | 1 (1.1) | 1 (0.6) | 4 (2.2) | 7 (5.1) | 0.060 |
| Chronic Pulmonary Disease (%) | 5 (5.5) | 15 (9.4) | 31 (17.1) | 11 (8.0) | 0.009 |
| Diabetes (%) | 24 (26.4) | 57 (35.8) | 70 (38.7) | 49 (35.8) | 0.249 |
| Hepatobiliary disease (%) | 17 (18.7) | 41 (25.8) | 38 (21.0) | 24 (17.5) | 0.325 |
| Renal Disease (%) | 8 (8.8) | 9 (5.7) | 16 (8.8) | 32 (23.4) | <0.001 |
| Malignant Tumor (%) | 1 (1.1) | 3 (1.9) | 7 (3.9) | 0 (0.0) | 0.086 |
| ICU Mortality (%) | 8 (8.8) | 0 (0.0) | 20 (11.0) | 4 (2.9) | <0.001 |
| Hospital Mortality (%) | 13 (14.3) | 1 (0.6) | 23 (12.7) | 6 (4.4) | <0.001 |

Table S3. The complete clinical characteristics of subphenotypes in validation cohort

|  | All Patients (3,180) | Phenotype A (460) | Phenotype B (620) | Phenotype C (887) | Phenotype D (1,213) |
| --- | --- | --- | --- | --- | --- |
| Hepatobiliary disease (%) | 37.3 | 34.8 | 35.6 | 32.9 | 42.3 |
| Diabetes (%) | 23.6 | 23.7 | 25.3 | 24.7 | 22.0 |
| Renal Disease (%) | 8.2 | 10.7 | 7.3 | 9.2 | 7.1 |
| Congestive Heart Failure (%) | 7.4 | 9.3 | 5.3 | 9.7 | 6.0 |
| Malignant Tumor (%) | 6.5 | 2.8 | 2.7 | 3.6 | 12.0 |
| Chronic Pulmonary Disease (%) | 5.8 | 3.5 | 5.2 | 6.0 | 6.8 |
| Cerebrovascular Disease (%) | 3.1 | 3.3 | 2.7 | 3.2 | 3.1 |
| Myocardial Infarction (%) | 1.2 | 0.4 | 0.8 | 1.5 | 1.5 |

Table S4. Fit statistics of GBMTM from 1 to 5 subclasses

| Model | Number of Class | Loglik | AIC | BIC | ICL | Entropy | % Class 1 | % Class 2 | % Class 3 | % Class 4 | % Class 5 | % Class 6 |
| --- | --- | --- | --- | --- | --- | --- | --- | --- | --- | --- | --- | --- |
| GBMTM-2 | 2 | -77351.6 | 154785.3 | 155088.5 | 155252.5 | 0.91 | 40.3 | 59.7 | / | / | / | / |
| GBMTM-3 | 3 | -75172.9 | 150928.3 | 150928.3 | 151239.1 | 0.89 | 49.3 | 27.1 | 23.6 | / | / | / |
| GBMTM-4 | 4 | -73283.5 | 146732.9 | 147346.9 | 147731.4 | 0.89 | 14.1 | 17.6 | 27.0 | 41.3 | / | / |
| GBMTM-5 | 5 | -72401.6 | 145011.2 | 145780.5 | 146235.4 | 0.89 | 17.4 | 17.9 | 9.8 | 38.4 | 16.5 | / |
| GBMTM-6 | 6 | -71573.3 | 143396.5 | 144321.2 | 144849.3 | 0.88 | 8.3 | 14.7 | 12.6 | 13.0 | 20.9 | 30.5 |

Loglik: Log-likelihood; AIC: Akaike information criterion; BIC: Bayesian information criteria; ICL: Integrated Completed Likelihood Criterion.

The model metrics of the GBMTMs reveal a specific pattern. As the value of K increases, we observe that the AIC, BIC, and ICL values of the model consistently decrease, indicating improved model performance. Additionally, the entropy measure remains consistently above 0.85, suggesting a robust model fit. This decline in AIC, BIC, and ICL reflects a better representation of the underlying data structure as K increases.

However, a critical observation arises when K reaches 5 or more: the smallest subgroup's proportion falls below 10%. This indicates that, although the model continues to fit the data well, it does so by dividing the data into increasingly smaller subgroups, which can complicate the interpretability of the results.

Considering these factors, K=4 emerges as the most optimal choice. This selection balances model fit, complexity, and subgroup size, providing a practical compromise that effectively captures the data's inherent structure without sacrificing interpretability.

Table S5. Fit Fixed effects of GBMTMs with 4 subclasses

| Class | Vital Sign | Intercept | Hour | (Hour)^2 |
| --- | --- | --- | --- | --- |
| 1 | SBP | -0.198 | -0.016 | 0.002 |
| 1 | DBP | -0.127 | -0.050 | 0.004 |
| 1 | HR | 1.488 | -0.066 | 0.001 |
| 1 | RR | 1.237 | -0.122 | 0.005 |
| 1 | TEMP | 0.454 | 0.175 | -0.013 |
| 2 | SBP | 1.187 | -0.126 | 0.007 |
| 2 | DBP | 1.174 | -0.106 | 0.006 |
| 2 | HR | 0.489 | -0.078 | 0.004 |
| 2 | RR | 0.655 | -0.087 | 0.003 |
| 2 | TEMP | -0.077 | 0.101 | -0.006 |
| 3 | SBP | -0.237 | -0.108 | 0.008 |
| 3 | DBP | -0.315 | 0.086 | 0.006 |
| 3 | HR | 0.130 | -0.045 | 0.002 |
| 3 | RR | 0.108 | -0.063 | 0.003 |
| 3 | TEMP | -0.333 | 0.103 | -0.004 |
| 4 | SBP | 0.409 | -0.126 | 0.007 |
| 4 | DBP | 0.369 | -0.102 | 0.005 |
| 4 | HR | -0.672 | -0.007 | 0.004 |
| 4 | RR | -0.715 | 0.017 | -0.001 |
| 4 | TEMP | -0.907 | 0.028 | 0.000 |

Table S6. Impact of comorbidities on 30-day mortality across

| Comorbidity | Phenotype A | | Phenotype B | | Phenotype C | | Phenotype D | |
| --- | --- | --- | --- | --- | --- | --- | --- | --- |
|  | HR(95%CI) | P-value | HR(95%CI) | P-value | HR(95%CI) | P-value | HR(95%CI) | P-value |
| Hepatobiliary disease | 0.80(0.42, 1.50) | 0.480 | 1.00(0.39, 3.12) | 0.859 | 1.05(0.61, 1.78) | 0.866 | 0.73(0.30, 1.77) | 0.488 |
| Diabetes | 1.01(0.50, 2.06) | 0.958 | 0.57(0.15, 2.11) | 0.397 | 1.53(0.89, 2.61) | 0.124 | 1.53(0.62, 3.79) | 0.352 |
| Renal Disease | 1.37(0.55, 3.42) | 0.501 | 1.90(0.43, 8.51) | 0.400 | 2.11(1.09, 4.08) | 0.026 | 3.72(1.32, 10.49) | 0.013 |
| Congestive Heart Failure | 0.67(0.22, 2.01) | 0.472 | 1.02(0.13, 8.01) | 0.982 | 0.79(0.32, 5.43) | 0.563 | 1.93(0.63, 5.88) | 0.245 |
| Malignant Tumor | 1.90(0.46, 7.95) | 0.375 | 6.50e-08(0, Inf) | 0.998 | 1.31(0.32, 5.43) | 0.708 | 1.13(0.26, 4.94) | 0.871 |
| Chronic Pulmonary Disease | 2.07(0.62, 6.89) | 0.233 | 5.16(1.10, 24.16) | 0.037 | 3.03(1.43, 6.40) | 0.003 | 0.68(0.09, 5.17) | 0.706 |

Table S7. Association between lactated Ringer’s use within 48 hours and 30-day mortality risk

| Lactated Ringer‘s Used in 48 Hours | Phenotype A | | Phenotype B | | Phenotype C | | Phenotype D | |
| --- | --- | --- | --- | --- | --- | --- | --- | --- |
|  | HR(95%CI) | P-value | HR(95%CI) | P-value | HR(95%CI) | P-value | HR(95%CI) | P-value |
| Crude | 0.53(0.29, 0.97) | 0.039 | 0.25(0.06, 1.09) | 0.060 | 0.47(0.28, 0.79) | 0.005 | 0.45(0.19, 1.03) | 0.058 |
| Adjusted | 0.66(0.35, 1.25) | 0.205 | 0.27(0.06, 1.22) | 0.089 | 0.48(0.28, 0.82) | 0.007 | 0.55(0.24, 1.31) | 0.178 |

Table S8. Missing data of clinical characteristics in development and validation cohorts

| Variable | Development Cohort | Validation Cohort |
| --- | --- | --- |
| Age | 0 | 0 |
| Gender | 0 | 0 |
| Race | 0 | 0 |
| Systolic Blood Pressure | 0 | 0 |
| Diastolic Blood Pressure | 0 | 0 |
| Heart Rate | 0 | 0 |
| Repatriation Rate | 0 | 0 |
| Temperature | 0 | 0 |
| Oxygen Saturation | 3.3 | 0.4 |
| Amylase | 15.5 | 30.1 |
| Lipase | 16.7 | 11.5 |
| Blood Urine Nitrogen | 1.1 | 0.6 |
| Creatinine | 1 | 0.4 |
| Sodium | 1 | 0 |
| Potassium | 1.5 | 0 |
| Calcium | 1.5 | 0.9 |
| Phosphate | 1.5 | 13.2 |
| White Blood Cell | 1.0 | 0.5 |
| C-Reactive Protein | 19.1 | 90.5 |
| Procalcitonin | 19.8 | Not Included |
| Hemoglobin | 1.0 | 0.5 |
| Hematocrit | 1.0 | 0.5 |
| pH | 3.3 | 19.8 |
| Glucose | 0.9 | 1.58 |
| Triglycerides | 1.3 | 51.6 |
| Fluid Intake 1^st^ Day | 0 | 0 |
| Fluid Intake 2^nd^ Day | 0 | 0 |
| ICU Length of Stay | 0 | 0 |
| Hospital Length of Stay | 0 | 0 |
| Myocardial Infarction | 0 | 0 |
| Congestive Heart Failure | 0 | 0 |
| Cerebrovascular Disease | 0 | 0 |
| Chronic Pulmonary Disease | 0 | 0 |
| Diabetes | 0 | 0 |
| Hepatobiliary disease | 0 | 0 |
| Renal Disease | 0 | 0 |
| Malignant Tumor | 0 | 0 |
| ICU Mortality | 0 | 0 |
| Hospital Mortality | 0 | 0 |

The proportion of missing data in the developmental and validation cohorts was calculated after including and excluding participants; The number in red color represented variables with missing rate > 20% or not contained in database, which will not be included for further analysis. The rate of missing data for procalcitonin in the validation cohort was 100% because procalcitonin was not recorded in the relevant data sources.

Table S9. list of dependent packages in R environment

| R package | Function | Version | Linkking |
| --- | --- | --- | --- |
| caret | Framework for machine learning model training | 6.0-94 | https://cran.r-project.org/package=caret |
| flexmix | Group based multivariable trajectory model(GBMTM) consturction | 2.3-19 | https://CRAN.R-project.org/package=flexmix |
| longmixr | Consensus Clustering to identify optimal number of clusters of GBMTM | 1.0.0 | https://cran.r-project.org/package=longmixr |
| mice | Multivariate imputation for missing data | 3.16.0 | https://cran.r-project.org/package=mice |
| pdp | framework for constructing partial dependence | 0.8l1 | https://cran.r-project.org/package=pdp |
| randomForest | Depending package for random forest classification model construction | 4.7-1.1 | https://cran.r-project.org/package=randomForest |
| survival | Cox proportional hazards regression model consturction |  | https://cran.r-project.org/package=survival |
| survminer | Plotting adjusted Kaplan-Meier analysis | 0.4.9 | https://cran.r-project.org/package=survminer |

All data analyses were performed under R environment (version 4.3.1)
